# Taurine activates the AKT–mTOR axis to restore muscle mass and contractile strength in human 3D *in vitro* models of steroid myopathy

**DOI:** 10.1242/dmm.050540

**Published:** 2024-04-24

**Authors:** Sheeza Mughal, Maria Sabater-Arcis, Ruben Artero, Javier Ramón-Azcón, Juan M. Fernández-Costa

**Affiliations:** ^1^ Institute for Bioengineering of Catalonia (IBEC), The Barcelona Institute of Science and Technology (BIST), C/Baldiri Reixac 10-12, E08028 Barcelona, Spain; ^2^ University Institute for Biotechnology and Biomedicine (BIOTECMED), University of Valencia, Dr Moliner 50, E46100 Burjassot, Valencia, Spain; ^3^ Translational Genomics Group, Incliva Health Research Institute, Dr Moliner 50, E46100 Burjassot, Valencia, Spain; ^4^ Joint Unit Incliva- CIPF, Dr Moliner 50, E46100 Burjassot, Valencia, Spain; ^5^ Institució Catalana de Reserca i Estudis Avançats (ICREA), Passeig de Lluís Companys, 23, E08010 Barcelona, Spain

**Keywords:** 3D bioengineered skeletal muscle tissues, Corticosteroids, Steroid myopathy, Taurine

## Abstract

Steroid myopathy is a clinically challenging condition exacerbated by prolonged corticosteroid use or adrenal tumors. In this study, we engineered a functional three-dimensional (3D) *in vitro* skeletal muscle model to investigate steroid myopathy. By subjecting our bioengineered muscle tissues to dexamethasone treatment, we reproduced the molecular and functional aspects of this disease. Dexamethasone caused a substantial reduction in muscle force, myotube diameter and induced fatigue. We observed nuclear translocation of the glucocorticoid receptor (GCR) and activation of the ubiquitin–proteasome system within our model, suggesting their coordinated role in muscle atrophy. We then examined the therapeutic potential of taurine in our 3D model for steroid myopathy. Our findings revealed an upregulation of phosphorylated AKT by taurine, effectively countering the hyperactivation of the ubiquitin–proteasomal pathway. Importantly, we demonstrate that discontinuing corticosteroid treatment was insufficient to restore muscle mass and function. Taurine treatment, when administered concurrently with corticosteroids, notably enhanced contractile strength and protein turnover by upregulating the AKT–mTOR axis. Our model not only identifies a promising therapeutic target, but also suggests combinatorial treatment that may benefit individuals undergoing corticosteroid treatment or those diagnosed with adrenal tumors.

## Teaser

**Summary:** A 3D muscle model reveals the therapeutic potential of taurine in countering steroid myopathy and enhancing muscle function, and suggests a combined therapeutic approach for patients on corticosteroids or with adrenal tumors.

## INTRODUCTION

Skeletal muscles are the largest and most adaptable protein reservoirs with a capacity to regulate their mass, protein turnover and fiber cross-sectional area in response to a variety of external and internal stimuli (Ferreira and Duarte, 2023). A well-regulated balance between proteolytic and proteogenic pathways is essential to maintain healthy muscular function (Vélez et al., 2017). During muscular atrophy, proteolytic pathways are hyperactivated, which results in a low protein turnover, a steep decline in muscle mass and loss of contractile function (Bonaldo and Sandri, 2013).

Excessive loss of muscle mass is also associated with poor disease prognosis (Bonaldo and Sandri, 2013). This holds particularly true for anti-inflammatory treatment protocols requiring extended use of oral or intravenous corticosteroids. Corticosteroid-induced muscular atrophy, also called glucocorticoid or steroid myopathy, is a toxic, iatrogenic, noninflammatory condition arising due to prolonged glucocorticoid use (Pereira and Freire de Carvalho, 2011). It is known to manifest through catabolic, anti-anabolic or both routes. Contrary to other muscular myopathies, in patients with steroid myopathy, standard diagnostic biomarkers such as creatine kinase, aspartate aminotransferase, lactate dehydrogenase and aldolase levels are often normal in the early disease stages. These markers might only be elevated in critically ill patients, making it difficult to get a timely diagnosis (Nagpal and Tierney, 2023).

Initially discovered in patients being treated for Cushing's disease with corticosteroids, steroid myopathy proceeds through hyperactivation of the ubiquitin–proteasome pathway (Pereira and Freire de Carvalho, 2011; Schakman et al., 2008). Muscle atrophy F-box protein (MAFbx, also known as atrogin-1 or FBXO32) and muscle-specific RING finger protein 1 (MURF1, also known as TRIM63) are two E3 ubiquitin ligases upregulated in the atrophying muscle. Therefore, these proteins are important markers in studying muscle mass regulation due to corticosteroid or glucocorticoid exposure (Bodine and Baehr, 2014).

The glucocorticoid receptor (GCR) gene (*NR3C1*, NM_000176.3) is a ligand-dependent transcription factor belonging to the nuclear receptor superfamily (Oakley and Cidlowski, 2013). It is expressed in all major cell types to enable the pleiotropic effects of glucocorticoids. In the absence of a ligand, the receptor is transcriptionally inactive and remains restricted in the cytoplasm as a multi-protein complex (Oakley and Cidlowski, 2013). This complex consists of chaperone proteins and immunophilins that regulate conformational change of GCR to enable high-affinity ligand binding and subsequent nuclear translocation (Grad and Picard, 2007). Prior to nuclear translocation, chaperone proteins dissociate, exposing two nuclear localization signals (NL1 and NL2). Once inside the nucleus, the central DNA-binding domain of GCR binds to glucocorticoid-responsive elements (GREs). These elements are imperfect palindromic sequences that allow GCR dimerization and subsequent transcription of multiple genes by recruiting RNA polymerase II (Braun and Marks, 2015).

GCR can initiate catabolic effects by inducing the expression of forkhead transcription factor O1 (*FOXO1*), which upregulates *FBXO32* and *TRIM63*, thereby upregulating protein catabolism in the muscle (Baehr et al., 2011; Bonaldo and Sandri, 2013). The anti-anabolic effects of GCR suppress the stimulatory effects of insulin, insulin-like growth factor I (IGF1) and amino acids on two key cellular components: eIF4E-binding protein 1 (4E-BP1 or EIF4EBP1) and ribosomal protein S6 kinase 1 (S6K1 or RPS6KB1) (Dos et al., 2004). These components directly regulate protein synthesis through the mammalian target of rapamycin (mTOR) pathway (Schakman et al., 2008). This exclusive and highly coordinated interaction between the catabolic and anabolic mechanisms is essential to understand how distortion of balance might impact muscle structural and functional capacity.

Amino acid supplementation is known to enhance isometric muscle strength and improve ambulatory dysfunction (Børsheim et al., 2008). The semi-essential micronutrient taurine is known for improving physical endurance by modulating excitation–contraction coupling in skeletal muscles and forms a crucial part of the cytoprotective mechanisms in the cell (Merckx and De Paepe, 2022; Schaffer et al., 2010; Scicchitano and Sica, 2018). According to Uozumi et al. (2006), skeletal muscle stores 70% of total taurine reserves in the body and the expression of taurine transporter (TauT or SLC6A6) is positively regulated under the myogenic program. In the same study, a higher muscular regeneration activity was also directly associated with higher taurine levels (Uozumi et al., 2006). The exact mechanism of the amelioration capacity of taurine for steroid myopathic skeletal muscle tissues, however, remains unexplored.

Although extensive research has been conducted to identify the mechanistic pathophysiology involved in muscular myopathies, contemporary research uses samples obtained from two-dimensional (2D) cell cultures or rodents. Not only do these procedures require exhaustive resources and provide no information on the *in situ* functional implications, but also quite often have limited reproducibility in humans (Mughal et al., 2022). Moreover, the percentage of US Food and Drug administration (FDA)-approved drugs to the monetary investment spent annually on drug development and validation studies continues to decrease monotonically. Three-dimensional (3D) *in vitro* tissues and organoids bioengineered from human-derived cells have garnered immense interest in mechanistic and pharmacological studies owing to their better reproducibility and similarity to the *in vivo* microenvironmental conditions (Fernández-Costa et al., 2023b; Ingber, 2022)

In this work, we developed 3D bioengineered skeletal muscle tissues to quantitatively map steroid myopathy. We used dexamethasone (DEX) to induce steroid myopathy and then studied the isolated and combined effect of the amelioration capacity of taurine. Although regulation of metabolism–volume coupling through glucocorticoids has been studied in mouse models and 2D cell cultures (Bodine and Baehr, 2014), there is presently no 3D model that relates the underlying molecular pathology to the functional capacity of a human muscle. Our 3D bioengineered skeletal muscle model for the first time replicated human steroid myopathy *in vitro*, particularly GCR translocation, and we used it as a drug-testing platform to study the mechanistic action of taurine.

Overall, our findings confirm that high systemic levels of DEX hyperactivate the ubiquitin–proteasomal degradation of the skeletal muscles. This causes a steep decline in maximum contractile force and an increase in fatigue. Moreover, upregulation of the phosphorylated (p-)AKT–p-mTOR axis by taurine in the diseased tissues not only promotes protein synthesis and restoration of functional and structural phenotypes, but does so possibly by inhibiting GCR recruitment onto the GREs through mTOR.

## RESULTS

### 3D bioengineered tissues atrophy following DEX treatment

**Fig. 1.**
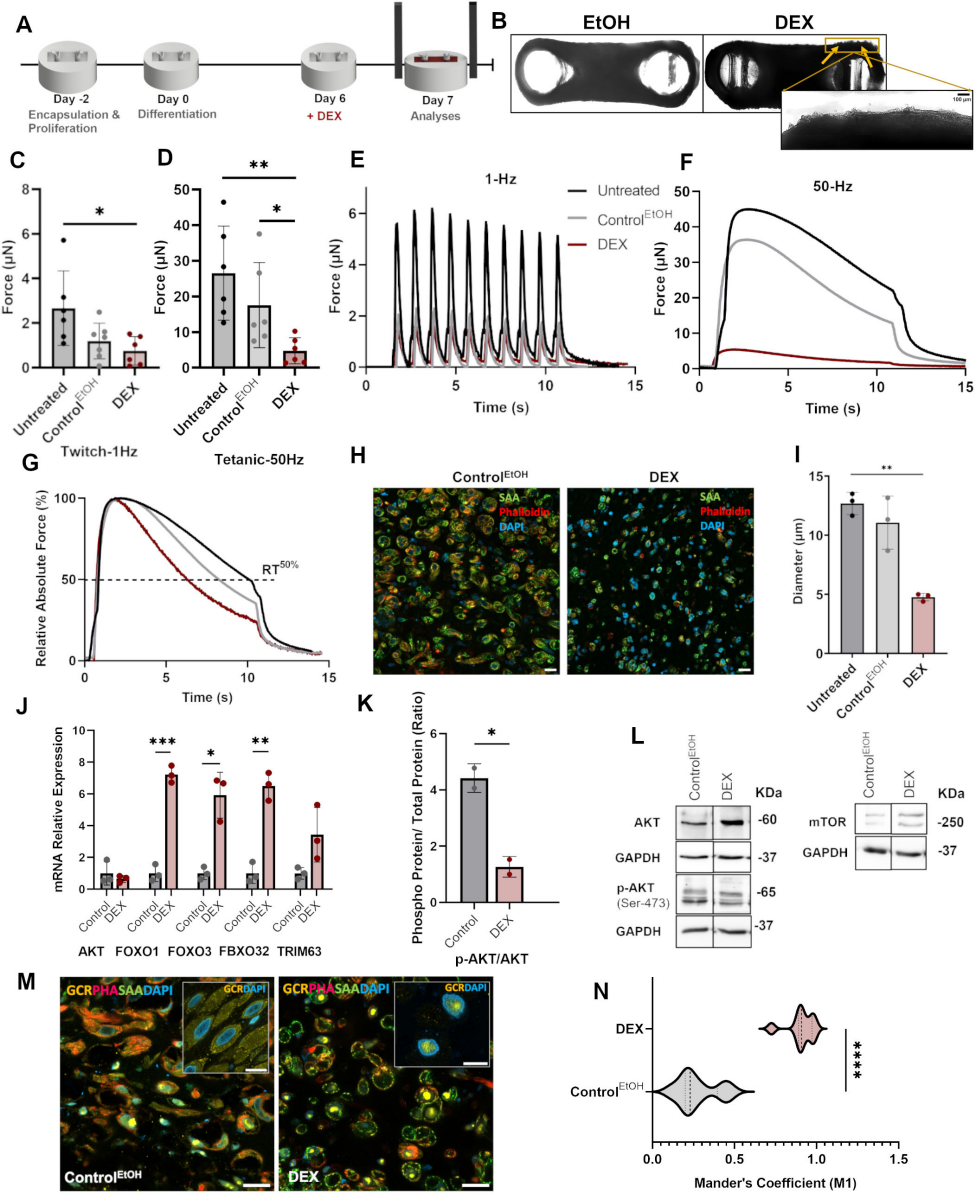
**3D *in vitro* bioengineered human skeletal muscle tissue model of steroid myopathy.** (A) Scheme of tissue fabrication and induction of steroid myopathy with dexamethasone (DEX). (B) Representative brightfield images of the top view of 3D skeletal muscle tissues treated with ethanol (EtOH, control) or DEX. Arrows point to broken myotubes. Scale bar (inset): 100 µm. (C,D) Maximum twitch (C) and tetanic (D) forces experienced by untreated, ethanol-treated or DEX-treated tissues. *n*=6 tissues per condition. (E) Comparative twitch spectrum at 1 Hz. (F) Comparative tetanic spectrum at 50 Hz. (G) Relative absolute force at 50 Hz. The dotted line indicates the time taken to reach 50% relaxation (RT^50%^). (H) Representative confocal images of transverse cross sections stained with an antibody against the mature muscle marker sarcomeric actinin (SAA), phalloidin (F-actin) and DAPI (nuclei). Scale bar: 20 µm. (I) Quantification of myotube diameters. *n*=3 tissues. (J) Relative expression of atrogene mRNAs determined by qRT-PCR. (K) Graph showing p-AKT_(Ser473)_/AKT_(total)_ protein ratios, determined by quantification of band intensities in western blots (*n*=2). (L) Representative western blots for AKT, p-AKT and mTOR, with GAPDH blots shown below as loading controls. The bands for the control and DEX-treated conditions are from the same membranes; uncropped blots are shown in Figs S7-S9. p-mTOR blots for these conditions and their quantification is not shown because the bands were on different membranes. These blots are shown in Fig. S10. (M) Representative confocal images of transverse cross sections stained with antibodies against SAA and glucocorticoid receptor (GCR), phalloidin (PHA) and DAPI. Scale bars: 20 µm and 10 µm (inset). The unmerged corresponding images are shown in Fig. S4A. (N) Mander's coefficients (M1) showing the colocalization of GCR with the nucleus. *n*=6. All experiments were repeated at least three times to confirm reproducibility. Data show the mean±s.d. Statistical analysis: one-way ANOVA with Tukey's post hoc test (C,D,I); two-tailed unpaired Student's *t*-test with Welch's correction (J,K,N). *P≤0.05; **P≤0.01; ***P≤0.001; ****P≤0.0001.

A basic hallmark of steroid myopathy is muscle weakness due to rapid proteolysis (Nagpal and Tierney, 2023). 3D *in vitro* skeletal muscle models provide an opportunity to study the functional and mechanistic implications of disease progression. To fabricate skeletal muscle 3D tissues, we encapsulated human myogenic precursor cells in a Matrigel–fibrin matrix. Before initiating differentiation, the cells were allowed to aggregate together to form essential cell–cell contacts. 3D *in vitro* skeletal muscle tissues had longitudinally aligned myofibers and the mature muscle marker sarcomeric actinin (SAA or ACTN2) (Fig. S1B,D), formed due to the passive tension of the pillars extending through the compacting matrix. All tissues derived from human myogenic precursor cells showed efficient matrix compaction post encapsulation, and longitudinally aligned, well-differentiated myofibers were observed stretching between the two pillars after 6 days (Fig. 1A). The hence-formed tissues were then treated with 100 µM DEX and ethanol for 24 h after 6 days of differentiation (Fig. 1A). We used ethanol as a control as it was the DEX diluent to rule out any changes due to effect of the vehicle. Prolonging the exposure of tissues to DEX for 48 h caused most of the tissues to break, therefore making it impossible to record their contractile profiles. All control tissues (treated with ≤1% of ethanol), and DEX-treated tissues were well compacted but some broken myotubes were observed at the margins for DEX-treated tissues. This could be because myotubes at the peripheral margins are more exposed to external stimuli than those deeply embedded in the center (Fig. 1B). 100 µM DEX-treated tissues exhibited a lower maximum force of contraction compared to that experienced by untreated and ethanol-treated controls. The same trend was observed for both twitch and tetanic stimulations (Fig. 1C-F). Twitch or isolated contractions are induced by exposure to low-frequency (1-2 Hz) pulses. On the contrary, tetanic contractions are sustained contractions at higher frequencies (25-50 Hz), which help indicate the maximum contractile force of the muscle (Movie 1). An electrical pulse stimulation scheme incorporating both types of frequencies allowed us to study the differential contraction dynamics of the tissues in response to different drugs (Fig. S1D). The difference in maximum contractile force between ethanol-treated controls and untreated tissues was not statistically significant. Nevertheless, the drop in strength observed for ethanol-treated samples indicates that although minimal, the toxic effect exists. A drop in strength or force of contraction in skeletal muscles is a direct indicator of a dysregulated volume–mass ratio, which compromises fiber contractile properties (Shimizu et al., 2011). The data obtained from electrical pulse stimulation indicated fatigue in the DEX-treated tissues, which is another characteristic of a weak contractile apparatus (Fig. 1G). The significant difference in the time taken to reach 50% relaxation between the myopathic and control tissues is further quantified in Fig. S3A. A decrease in myotube diameter for myopathic tissues, quantified through immunofluorescence, further corroborated this trend (Fig. 1H,I). Subsequent analysis of GCR translocation in the nucleus showed a higher internalization of the receptor in DEX-treated tissues compared to that in the controls (Fig. 1M; Fig. S4A), as indicated by Mander's coefficient (M1) (Fig. 1N), which measured the colocalization of GCR immunofluorescence signals with DAPI fluorescence signals.

To further analyze these signs of atrophy due to DEX, we quantified the mRNA levels of atrogenes. Quantitative real-time PCR (qRT-PCR) data of the expression of atrogenes implicated in the IGF1R–AKT–FOXO pathway indicated elevated levels of *FOXO1* and *FOXO3* RNAs in DEX-treated tissues compared to those in controls (Fig. 1J). Both *FOXO1* and *FOXO3* have GREs, enhancing gene transcription significantly. Protein kinase B (*AKT* or *AKT1*) transcript levels, however, remained the same in all treatment conditions, confirming that glucocorticoids do not affect genetic transcription of *AKT*. *TRIM63* contains GREs but *FBXO32* does not.

To investigate the AKT pathway, we performed western blot analyses to quantify alterations at the protein level. The results indicated that the p-AKT_(Ser473)_/AKT_(total)_ ratio decreased in DEX-treated tissues (Fig. 1K,L), which explains the observed increase in FOXO-dependent *FBXO32* and *TRIM63* transcription. Interestingly, these findings indicate both catabolic and anti-anabolic routes of atrophy induction. Although DEX upregulated both E3 ubiquitin ligases – *FBXO32* by increasing the levels of FOXO transcription factors only, and *TRIM63* through both FOXO-responsive elements and GREs – it downregulated p-AKT levels, by upregulating *REDD1* and *KLF15* as demonstrated previously (Shimizu et al., 2011). This axis then regulates the muscle mass and the contractile profile of the muscle as well. The protocol, therefore, successfully induced steroid myopathy in 3D *in vitro* human skeletal muscle tissues.

### DEX replacement with taurine partially restores functional capacity

**Fig. 2.**
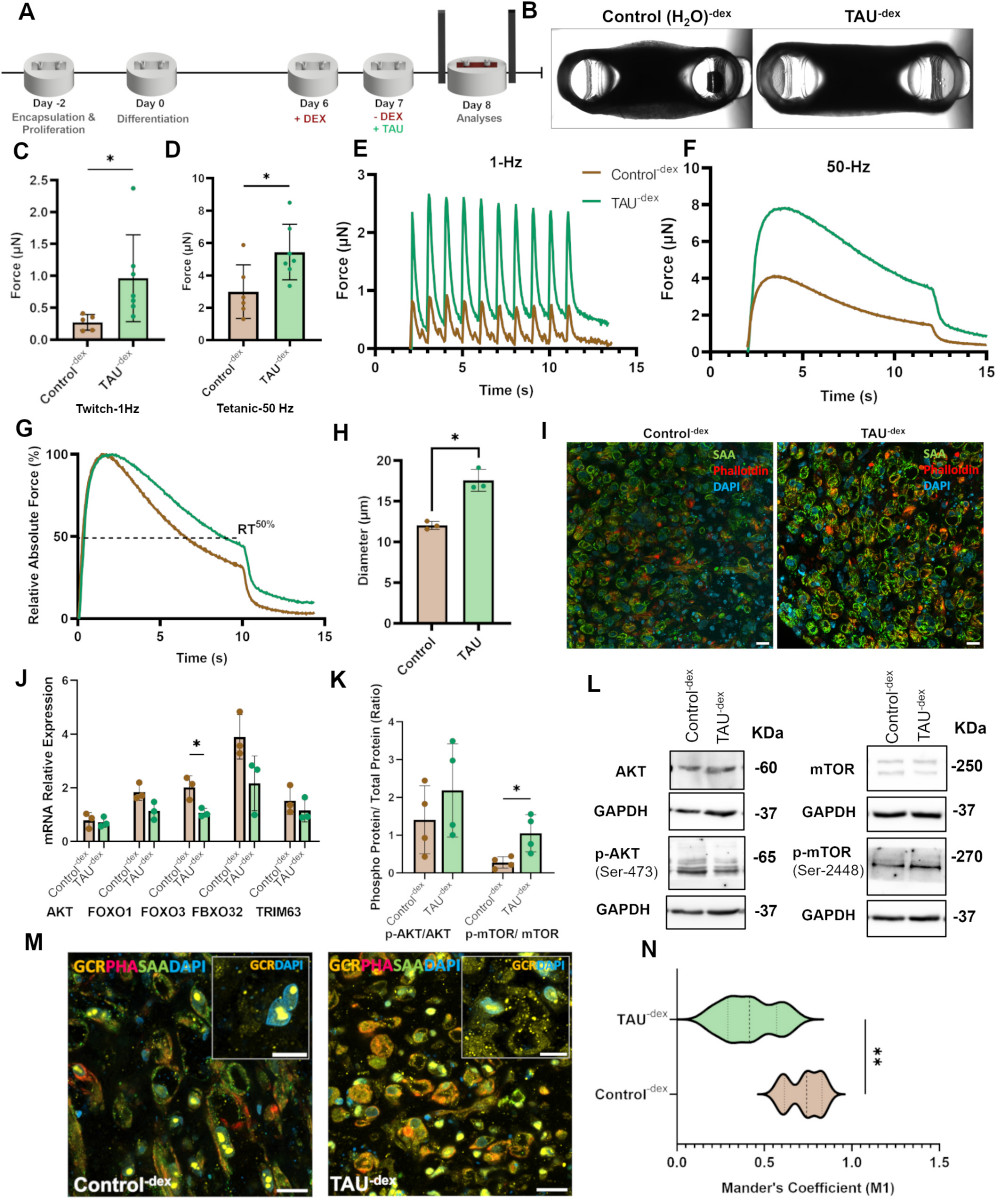
**Effect of taurine on DEX-treated tissues.** (A) Scheme of myopathy induction with dexamethasone (DEX) and taurine (TAU) treatment without DEX. (B) Representative brightfield images of the top view of 3D skeletal muscle tissues for the indicated treatment conditions. (C,D) Maximum twitch (C) and tetanic (D) forces experienced by tissues. *n*=6 tissues per condition. (E) Comparative twitch spectrum at 1 Hz. (F) Comparative tetanic spectrum at 50 Hz. (G) Relative absolute force at 50 Hz. The dotted line indicates the time taken to reach 50% relaxation (RT^50%^). (H) Quantification of myotube diameters for the indicated conditions. *n*=3 tissues. (I) Representative confocal images of transverse cross sections stained with an antibody against sarcomeric actinin (SAA), phalloidin (F-actin) and DAPI (nuclei). Scale bar: 20 µm. (J) Relative expression of atrogene mRNAs determined by qRT-PCR. (K) Graph showing p-AKT_(Ser473)_/AKT_(total)_ and p-mTOR_(Ser2448)_/mTOR_(total)_ protein ratios, determined by quantification of band intensities in western blots (*n*=4). (L) Representative western blots for AKT, p-AKT, mTOR and p-mTOR, with GAPDH blots shown below as loading controls. (M) Representative confocal images of transverse cross sections stained with antibodies against SAA and glucocorticoid receptor (GCR), phalloidin (PHA) and DAPI. Scale bars: 20 µm and 10 µm (inset). The unmerged corresponding images are shown in Fig. S4B. (N) Mander's coefficient (M1) showing the colocalization of GCR with the nucleus. *n*=6. All experiments were repeated at least three times to confirm reproducibility. Data show the mean±s.d. Statistical analysis: two-tailed unpaired Student’s *t*-test with Welch's correction (C,D,H,J,K,N). *P≤0.05; **P≤0.01.

To check the restoration capacity of taurine for tissues with steroid myopathy, we completely removed glucocorticoid-supplemented medium from the tissues after 24 h and replaced it with fresh medium containing either MilliQ water (control) or 1 mM taurine (Fig. 2A). The tissues were maintained in the new medium for 24 h before analyses. Changing the medium did not affect tissue compaction as clearly observed in Fig. 2B. Removal of DEX in both cases improved force and myotube diameter overall (Fig. 2C-F,H,I). The restoration capacity of taurine was, however, significantly higher compared to that in the control (Fig. 2C-F,H,I). Moreover, the control tissues indicated fatigue, calculated by the time taken to achieve 50% relaxation (Fig. 2G; Fig. S3B). Comparison of the nuclear translocation of GCR indicated a significantly higher GCR localization in the nucleus of control tissues compared to that in taurine-treated tissues (Fig. 2M,N; Fig. S4B).

To check whether taurine induced any effects at the transcription level, qRT-PCR was performed to check for any changes in the ubiquitin–proteasome pathway. qRT-PCR of mRNA showed similar levels of *AKT* in taurine-treated and control tissues (Fig. 2J). A partial decrease in *FOXO1* and significant decrease in *FOXO3* levels were observed in favor of taurine supplementation. The levels of *TRIM63* and *FBXO32* decreased overall but remained partially different between the two groups. The data suggest that as long as glucocorticoids remain in the system, *FOXO1* and *FOXO3* transcription is promoted and, consequently, *FBXO32* mRNA levels will increase. Removal of glucocorticoids was not sufficient to restore, even partially, the contractile strength and protein turnover in the myopathic tissues. Supplementation with taurine helped to significantly reverse the damage inflicted by 24-h glucocorticoid treatment compared to that in control tissues by significantly reducing the levels of *FOXO3*, which partially reduced *FBXO32* transcription.

At the protein level, the p-AKT_(Ser473)_/AKT_(total)_ ratio improved for taurine-supplemented tissues. Moreover, p-mTOR levels were significantly higher in the taurine-supplemented group (Fig. 2K,L). mTOR is a nutritional sensor in skeletal muscles known to regulate hypertrophy and muscle mass. These protein levels indicate that taurine upregulates p-AKT and p-mTOR and promotes protein synthesis while downregulating the transcription of atrogenes in the steroid myopathic human skeletal muscle tissues. The findings also substantiate that only replenishing with fresh medium and removing glucocorticoids from the system is insufficient to restore protein synthesis in myopathic tissues. Although the removal of glucocorticoids may help in reducing the transcription of atrogenes, it is not sufficient for the restoration of protein synthesis cascades through the AKT pathway.

### The restoration capacity of taurine is more pronounced with DEX in the medium

**Fig. 3.**
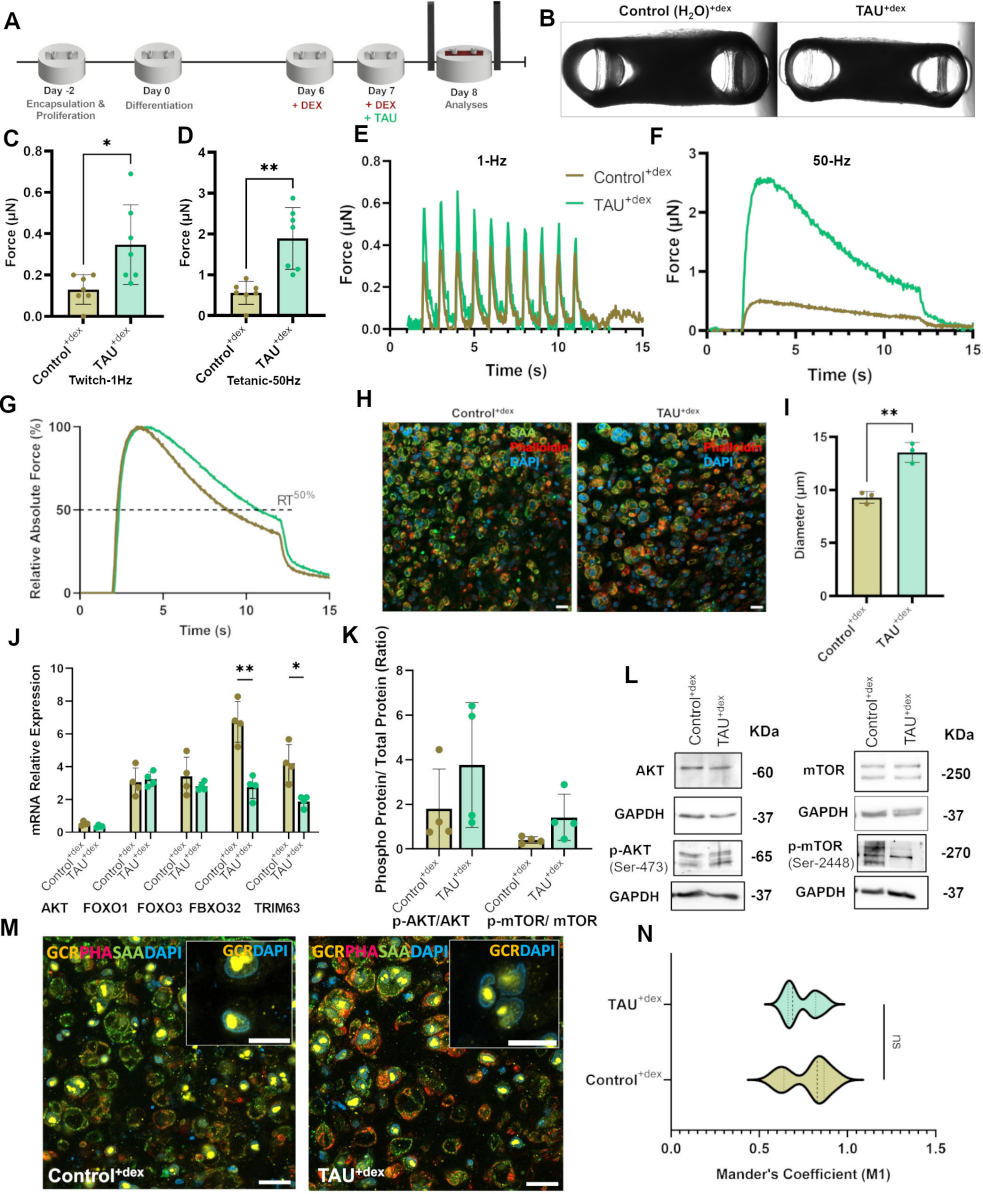
**Effect of taurine treatment concurrently with DEX.** (A) Scheme of myopathy induction with DEX and taurine (TAU) treatment with DEX. (B) Representative brightfield images of the top view of 3D skeletal muscle tissues for the indicated treatment conditions. (C,D) Maximum twitch and tetanic (D) forces experienced by tissues. *n*=6 tissues per condition. (E) Comparative twitch spectrum at 1 Hz. (F) Comparative tetanic spectrum at 50 Hz. (G) Relative absolute force at 50 Hz. The dotted line indicates the time taken to reach 50% relaxation (RT^50%^). (H) Representative confocal images of transverse cross sections stained with an antibody against sarcomeric actinin (SAA), phalloidin (F-actin) and DAPI (nuclei). Scale bar: 20 µm. (I) Quantification of myotube diameters. *n*=3 tissues. (J) Relative expression of atrogene mRNAs determined by qRT-PCR. (K) Graph showing p-AKT_(Ser473)_/AKT_(total)_ and p-mTOR_(Ser2448)_/mTOR_(total)_ protein ratios, determined by quantification of band intensities in western blots (*n*=4). (L) Representative western blots for AKT, p-AKT, mTOR and p-mTOR, with GAPDH blots shown below as loading controls. (M) Representative confocal images of transverse cross sections stained with antibodies against SAA and glucocorticoid receptor (GCR), phalloidin (PHA) and DAPI. Scale bars: 20 µm and 10 µm (inset). The unmerged corresponding images are shown in Fig. S4C. (N) Mander's coefficient (M1) showing the colocalization of GCR with the nucleus. *n*=6. All experiments were repeated at least three times to confirm reproducibility. Data show the mean±s.d. Statistical analysis: two-tailed unpaired Student’s *t*-test with Welch's correction (C,D,I,J,N). ns, not significant; *P≤0.05; **P≤0.01.

We checked the ability of taurine to competitively regulate protein turnover and contractile properties with glucocorticoids in the system (Fig. 3A). Treating myopathic tissues with 1 mM taurine in the presence of 100 µM DEX did not have an effect on the shape of the tissues (Fig. 3B), but the tissues presented a significant restoration of contractile force for both tetanic and twitch contractions, compared to that in control tissues treated with 100 µM DEX and MilliQ water (Fig. 3C-F). The myotube diameter was also found to increase, along with an improvement in muscle fatigue (Fig. 3G-I; Fig. S3C). As demonstrated by Mander's coefficient (M1), taurine supplementation was not capable of expelling the glucocorticoid-GCR complex from the nucleus (Fig. 3M,N; Fig. S4C), confirming that taurine does not affect nuclear translocation of the receptor and that this translocation is predominantly driven by glucocorticoids in the system. *AKT* mRNA levels remained the same, as did the levels of *FOXO1* and *FOXO3* (Fig. 3J), which further confirmed that taurine does not directly affect the transcriptional regulation of these factors. Their transcription is solely driven by GREs, indicating that the presence of glucocorticoids in the system will keep the transcription of these factors ongoing. *TRIM63* and *FBXO32* expression levels were, however, significantly reduced with taurine in the medium, whereas the p-AKT_(Ser473)_/AKT_(total)_ protein ratio increased substantially, as did the p-mTOR_(Ser2448)_/mTOR_(total)_ protein ratio (Fig. 3K,L). These findings suggest that an upregulation of p-AKT and p-mTOR prevented GRE-mediated transcription of the two ubiquitin ligases. It appears that taurine activated AKT and mTOR with glucocorticoids still in the system, more so compared to treating myopathic tissues with no DEX in the system.

### Taurine raises p-AKT levels in healthy skeletal muscle tissues

**Fig. 4.**
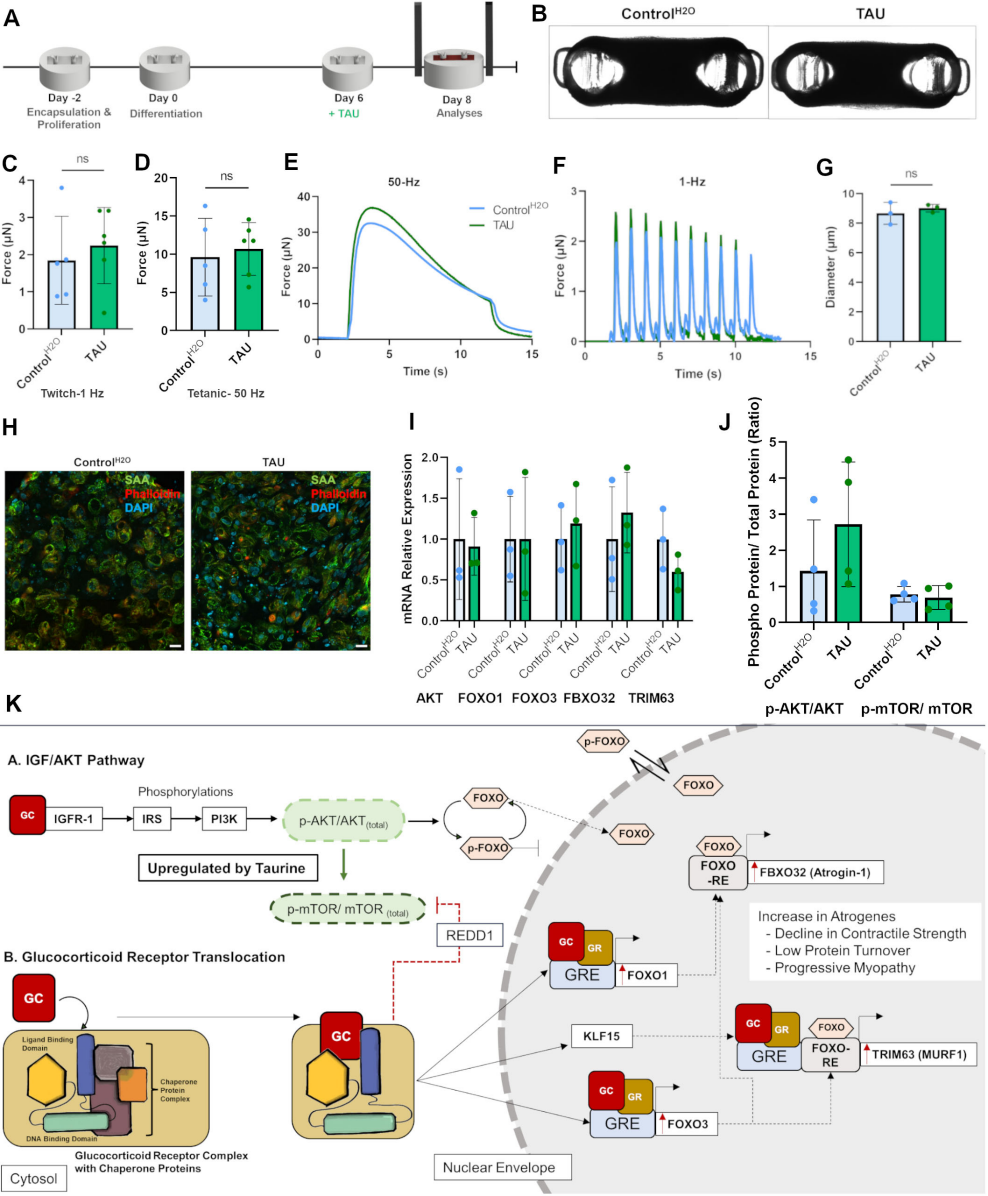
**Overall regulation of the IGF/AKT pathway by taurine.** (A) Scheme of taurine (TAU) treatment on healthy tissues. (B) Representative brightfield images of the top view of 3D skeletal muscle tissues for the indicated treatment conditions. (C,D) Maximum twitch (C) and tetanic (D) forces experienced by tissues. *n*=6 tissues per condition. (E) Comparative tetanic spectrum at 50 Hz. (F) Comparative twitch spectrum at 1 Hz. (G) Quantification of myotube diameters for the indicated conditions. *n*=3 tissues. (H) Representative confocal images of transverse cross sections stained with an antibody against sarcomeric actinin (SAA), phalloidin (F-actin) and DAPI. Scale bar: 20 µm. (I) Relative expression of atrogene mRNAs determined by qRT-PCR. (J) Graph showing p-AKT_(Ser473)_/AKT_(total)_ and p-mTOR_(Ser2448)_/mTOR_(total)_ protein ratios, determined by quantification of band intensities in western blots (*n*=4). The blots used for quantification are shown in Figs S7-S10. All experiments were repeated at least three times to confirm reproducibility. Data show the mean±s.d. Statistical analysis: two-tailed unpaired Student’s *t*-test with Welch's correction (C,D,G). ns, not significant. (K) Pathways implicated in glucocorticoid exposure and the mechanism of action of taurine. GC, glucocorticoid; GR, glucocorticoid receptor complex; RE, responsive element.

To validate our findings, we checked the effect of taurine on healthy tissues. After allowing the tissues to proliferate in growth medium for 2 days, the growth medium was changed to differentiation medium. On day 6 of differentiation, the tissues were treated with 1 mM taurine (Fig. 4A). All treated tissues formed well-compacted and differentiated tissues capable of contraction on electrical stimulation as observed under brightfield microscopy (Fig. 4B). Interestingly, taurine did not increase the force or induce hypertrophy in healthy tissues (Fig. 4C-H). No differences were observed between the controls and taurine-treated healthy tissues. Moreover, the RNA levels of atrogenes also remained at baseline, as did the levels of p-mTOR_(Ser2448)_/mTOR_(total)_ (Fig. 4I,J). Despite large sample variability, there was a substantial increase in the levels of p-AKT_(Ser473)_/AKT_(total)_, marking AKT phosphorylation as a direct target of the amino acid (Fig. 4J). For all the above treatments (including the myopathic tissues), total FOXO1 protein was found to localize within the cytoplasm (Fig. S2), perhaps due to the electrical pulse stimulation performed on all tissues prior to their fixation (poster by Rosales-Soto et al., 2023, http://hdl.handle.net/10713/20639). These findings highlight that taurine effectively regulates the AKT–mTOR axis through p-AKT in our *in vitro* bioengineered 3D skeletal muscle tissues to counter damage due to downregulation of the phosphorylation cascade and subsequent activation of the ubiquitin–proteasome system.

## DISCUSSION

An accurate identification of steroid myopathy relies on a strong clinical suspicion for patients with moderate to chronic exposure of corticosteroids. Elevated systemic corticosteroid levels might stem from excessive endogenous corticosteroid production caused by adrenal tumors, or due to an excess of exogenous steroid administration aimed at managing various health conditions such as asthma, chronic obstructive pulmonary disease (COPD) and rheumatoid arthritis (Nagpal and Tierney, 2023; Schakman et al., 2008). Steroid myopathy causes muscle weakness and wasting but without inducing any pain. There are no signs of inflammation and normal levels of creatine kinase, aldolase, aspartate aminotransferase and lactate dehydrogenase are found in the patients (Nagpal and Tierney, 2023). Abrupt corticosteroid withdrawal is the immediate response to stop disease progression and morbidity, but this approach does not help in restoring muscle strength.

Corticosteroids hinder the transport of amino acids into cells, suppress the production of muscle IGF1, and decrease the differentiation of precursor cells into muscle fibers (Pereira and Freire de Carvalho, 2011). As a result, this inhibition leads to reduced protein synthesis and impaired myogenesis. Contemporary literature points to the functional cooperativity between GCRs, GREs and FOXOs in regulating the expressions of atrogenes ,which eventually control the protein turnover of a muscle (Nagpal and Tierney, 2023; Pereira and Freire de Carvalho, 2011; Schakman et al., 2008; Shimizu et al., 2011). Although FOXOs have been singled out as major catabolic regulators, the direct and indirect influence of GCRs on the transcriptional regulation of atrogenes and mTOR-driven hypertrophic cascades cannot be ignored. Amino acid supplementation has been largely considered to control osmoregulation and protein stability within a muscle (Schaffer et al., 2010). However, their contribution to managing and countering signaling cascades warrants further investigation. Contextual to their contractile performance, it is essential to understand how the synchronization of atrophy pathways is orchestrated in skeletal muscle tissue and how, subsequently, balance is restored between protein synthesis and degradation. Through the application of a functional 3D muscle model, we reproduced the pathophysiology of steroid myopathy in human skeletal muscle tissue and subsequently investigated the mechanistic regulation of the nutritional sensor by taurine supplementation.

Tissues exposed to DEX underwent ubiquitin–proteasomal degradation as indicated by elevated levels of *TRIM63* and *FBXO32* through GCR-GRE and FOXO-mediated transcription. As a result, there was a significant drop in both tetanic and twitch contractile forces after 24 h of exposure. Prolonging the exposure to 48 h caused most of the tissues to break, thereby making it impossible to record their contractile profiles. In an atrophying skeletal muscle, there is a downregulation of the signaling cascade downstream of AKT. This permits de-phosphorylation of FOXO1 and FOXO3, allowing their translocation to the nucleus, subsequent attachment to FOXO-responsive elements and transcription of the E3 ubiquitin ligase genes *TRIM63* and *FBXO32* (Fig. 4K). A second pathway, driven primarily by the glucocorticoid receptor also operates simultaneously alongside AKT downregulation (Fig. 4K).

Once a glucocorticoid receptor bound with the ligand internalizes in the nucleus, it initiates transcription by binding to GREs (Oakley and Cidlowski, 2013). Although both *TRIM63* and *FBXO32* have FOXO-responsive elements, only the former has a GRE sequence. A significant increase in RNA levels was observed for *FBXO32* after 24-h treatment with DEX, but not for *TRIM63*, which increased after an additional 24-h exposure with DEX (Fig. 1J).

In addition to these atrogenes, GCR also induces transcription of *KLF15* and *REDD1*, which downregulate the signaling cascade of kinases controlled by mTOR, as apparent by the protein expression levels of mTOR in atrophying tissues (Shimizu et al., 2011). Besides controlling hypertrophy and protein synthesis, mTOR also suppresses glucocorticoid receptor-mediated transcription by inhibiting recruitment onto GREs (Yoon, 2017). Downregulation of both *AKT* and mTOR in conjunction with upregulation of *FOXO1* and *FOXO3* transcription disrupted protein turnover, hampered amino acid import, and induced proteolytic degradation in the myopathic myotubes, resulting in weaker and thinner tubes.

Although the induction of steroid myopathy via DEX has effectively produced myopathic tissues, it is imperative to acknowledge the potential impact of ethanol as a vehicle in this experimental design. As clearly observed (Fig. 1), there is a significant difference in all parameters between the control and DEX. Moreover, the dose-response curve for ethanol in muscle progenitor cells also indicated 100% viability at 1% or lower concentrations, whereas the toxic response increased beyond 5% (v/v) of ethanol (Fig. S5). Furthermore, evidence from the literature also suggests that lower concentrations of ethanol do not adversely impact muscle structure and performance (SIDS initial assessment report, 2004; Safety data sheet: Dexamethasone (with ethanol) formulation, Merck, 2023; Safety data sheet: Dexamethasone (with ethanol) formulation, MSD, 2023). Chronic use at higher concentrations, however, may have deleterious effects. *In vivo* studies undertaken in mice suggest atrophy at chronic dosages with at least 33% (v/v) ethanol consumption (Ni et al., 2013). Therefore, at concentrations of 1% (v/v) ethanol or lower, no deleterious effects of the vehicle can be attributed to our myopathic model.

Steroid myopathy was first discovered in a 24-year-old patient undergoing intravenous hydrocortisone treatment for asthma exacerbation (Pasnoor et al., 2014). Although the treatment cured her airway obstruction; her muscles were unable to support her weight. After the treatment was suspended, she was able to walk unassisted after 3-4 weeks but the weakness in her distal limb muscles persisted (Pasnoor et al., 2014). This proves that immediate withdrawal of corticosteroids is an effective emergency intervention but not sufficient to improve muscle function and structure long-term. The same was also observed in our model when we evaluated the response of our myopathic tissues to glucocorticoid-free medium. Although the contractile profile of these tissues improved, the effect was not significant. Moreover, the tissues were fatigued and lost strength, as evidenced by their reduced time to reach half relaxation. This quicker relaxation is symptomatic of a flaccid, weak tissue, which was further confirmed by thinner myotubes and elevated levels of *FBXO32*. The levels of *TRIM63*, however, remained the same as those in controls after medium replenishment. On the contrary, when myopathic tissues were treated with taurine-supplemented, glucocorticoid-free medium, we observed a significant improvement in contractile strength and myotube diameter compared to those in controls without taurine (Fig. 2). Upon further investigation, taurine was able to significantly lower the levels of *FOXO3* and there was a partial reduction in the transcription of other atrogenes. Phosphorylated protein levels of both AKT and mTOR increased, but the former was not significant compared to myopathic tissues in glucocorticoid-free medium. Complete cessation of glucocorticoid use with taurine supplementation can sufficiently improve muscle strength and protein turnover.

Complete removal of systemic corticosteroids is often not a practically viable solution (Merckx and De Paepe, 2022). In these cases, it is pivotal to not only provide symptomatic treatment to patients but also to check myopathic progression. To this end, after induction of myopathy, we kept the tissues in medium containing DEX during the treatments (Fig. 3A). Although glucocorticoids remained in the medium, taurine significantly not only rescued the functional and physiological parameters, but also significantly lowered the transcription of *FBXO32* and *TRIM63*. This was inferred to be a direct consequence of an increase in p-mTOR and p-AKT levels. p-AKT phosphorylated FOXO proteins and inhibited their translocation to the nucleus, whereas p-mTOR upregulated protein synthesis, induced myotube hypertrophy and restricted GCR attachment to the GREs, resulting in a twofold drop in the transcription levels of *FBXO32* and *TRIM63* (Fig. 3J), as previously reviewed by Yoon (2017).

It is pivotal to highlight that a corticosteroid treatment is often given to induce immunosuppression in patients with inflammatory conditions or those receiving an organ transplant; in such cases, it is undesirable to induce inflammation by inhibiting GCR recruitment to GREs. The anti-inflammatory potential of taurine has been demonstrated previously (Marcinkiewicz and Kontny, 2014; Qaradakhi et al., 2020; Schaffer and Kim, 2018; Terrill et al., 2020). As demonstrated in *mdx* mice models, not only is taurine non-inflammatory, but it also protects against neutrophil invasion in myotubes by scavenging HOCl produced by these immune cells to taurine chloramines (TauCls), which are less reactive than the former and prevent against the further production of pro-inflammatory cytokines (Terrill et al., 2020). Increase in the muscle levels of HOCl causes oxidative damage, compromising muscle strength and stability (Schaffer and Kim, 2018). The myeloperoxidase-catalyzed reaction contributes to the anti-inflammatory effects of taurine. TauCl, formed during this reaction, also mitigates an increase in nitric oxide and prostaglandin E2, reduces the activity of matrix metalloproteinases and triggers leukocyte apoptosis, thereby effectively countering acute inflammation (Marcinkiewicz and Kontny, 2014). It is, therefore, beneficial in the context of immunosuppression to have high taurine content not only in the myotubes, but also in the neutrophils. Owing to its established anti-inflammatory capacity, taurine is commonly used in patients with inflammatory conditions such as arthritis (Schaffer and Kim, 2018). In line with this contextual evidence, our model also indicates that although there is a marginal tendency due to the effect of mTOR upregulation, there is no significant reduction in the translocation of the GCR-GRE complex to the nucleus (Fig. 3M,N; Fig. S4C). Instead, taurine largely exerts its protective effects against steroid myopathy by regulating the AKT–mTOR axis through p-AKT upregulation (Fig. 4J).

Therefore, combinatorial therapy of glucocorticoids with taurine supplementation offers a more feasible solution to initiate protein synthesis in muscle tissues with steroid myopathy without having to clear the system of glucocorticoids. These results also indicate that a combinatorial therapy of glucocorticoids with taurine for patients undergoing treatment with steroids for a different health condition such as COPD, or only taurine in the case of adrenal tumors, can help protect patients against progressive myopathy. Furthermore, conclusions from the two rescue regimens also warrant in favor of the restoration capacity of taurine instead of an effect due to lowering steroid dosage. The first rescue experiment proves that when the medium containing DEX to induce damage was entirely replaced and taurine added, there was a significant restoration in force. The second treatment protocol, in contrast, included both DEX and taurine, again establishing the superior restoration capacity of taurine compared to that of the vehicle. This suggests that although minor improvement was observed upon lowering or even entirely removing DEX from the system, it was not as significant as when taurine is added.

To validate the mechanistic regulation by taurine, we checked whether isolated taurine treatment to healthy tissues brought any changes (Fig. 4). Interestingly, treating healthy skeletal muscle tissues with taurine did not increase contractile strength and average myotube diameter or change the levels of atrogenes. It did, however, substantially increase the protein levels of p-AKT.

Existing models of steroid myopathy use 2D cell cultures of C2C12 cells or study contractile profiles of muscles excised from mice (Baehr et al., 2011; Bonaldo and Sandri, 2013; Doss et al., 2022; Mughal et al., 2022). Although these models have immensely added to the existing knowledgebase, they cannot be completely relied upon to study disease progression and develop therapeutic interventions for humans due to their limited ability to reproduce biological complexity. For instance, data from 2D rodent cell culture demonstrate an increased expression of *TRIM63* levels but not of *FBOXO32* following DEX treatment of tissues (Shimizu et al., 2011). Yet this trend is reversed in our human precursor cell-derived 3D model.

3D *in vitro* skeletal muscle engineering has been used to fabricate models for a variety of complex diseases and has been proven to be an efficient platform for drug testing and identification of therapeutic targets (De Chiara et al., 2022; Fernández-Costa et al., 2023a; Fernández-Garibay et al., 2021; Fernández-Garibay et al., 2022). Our model not only identified mTOR and AKT as the key metabolic targets of muscle mass regulation by taurine, but also related taurine directly to the detailed regulation of contractile profiles for tissues with steroid myopathy. To the best of our knowledge, this is the first 3D skeletal muscle model derived from human muscle precursor cells that replicates steroid myopathy at the functional and molecular levels. It also explores the protective mechanism of taurine supplementation against steroid myopathy, thereby opening the door to exploring the therapeutic potential of taurine for patients facing a diagnosis of progressive steroid myopathy.

## MATERIALS AND METHODS

### Mold design and fabrication

Master molds for the 3D hydrogel casting platforms were designed using Fusion 360 by Autodesk (Fernández-Costa et al., 2023a). In this protocol, the final polydimethylsiloxane (PDMS) (10:1) molds were made using PDMS (5:1) intermediary negative molds (Fig. S1A).

#### 3D printing by digital light projection

SolusProto (Reify3D, CA, USA), an opaque high-temperature-resistant resin, was used to print master molds with a Direct Light Projection 3D printer (Solus DLP 3D Printer, Reify3D) at low resolution (80×45 mm with a pixel distance of 45 μm). The design was converted to a Standard Tri-angle Language (STL) format. Initial layer thickness was maintained at 30 µm and the light exposure time was set at 2 s per layer at an intensity of 7. A buffer time of 2 s allowed the platform to settle against turbulence before it continued moving downwards. After printing, the molds were thoroughly sonicated and washed with RESINAWAY (Monocure 3D, CA, USA) and MilliQ water and dried with nitrogen. The molds were then cured in ultraviolet light for 45 min. To remove any uncured resin from the casting cavity, the molds were placed for 1 h at 120°C on the hot plate (on an overturned glass Petri plate covered with an overturned big beaker to avoid cracking). Prior to silanization, the thermo-cured master molds were activated with ozone plasma for 30 s and silanized by chemical vapor deposition in a vacuum desiccator with a few drops of trichloro (1H,1H,2H,2H-perfluorooctyl)-silane (PFOTS, Sigma-Aldrich) (placed separately in an open Petri dish) for 1 h.

#### Fabrication of negative molds

The PDMS-negative mold was made by mixing the elastomer and curing agent (two parts polymer: base and curing agent, Sylgard 184, Dow Corning, MI, USA) (5:1). Prior to pouring on the master molds, the mixture was degassed in a vacuum desiccator to remove bubbles. After pouring, the molds were again degassed for 10 min in the desiccator to remove any entrapped bubbles and they were cured overnight at 75°C. Bubbles entrapped in the pillars were removed using a 0.41 mm dispensing tip syringe when needed. The negative mold was peeled and washed thoroughly with ethanol and MilliQ water, dried with nitrogen and silanized with a few drops of PFOTS (placed separately in an open Petri dish) for 1 h.

#### Fabrication of the PDMS platforms

The final PDMS platforms were made by mixing the elastomer and curing agent in a ratio of 10:1. The mixture was thoroughly mixed and degassed for 10-15 min at 0.7 MPa vacuum pressure. The uncured PDMS mixture was poured on the silanized negative molds and placed in a plastic Petri dish. The molds were again degassed to remove any trapped bubbles. For curing, the molds were left at 75°C overnight covered with a glass slide to achieve a smooth surface. After demolding, the PDMS platforms were obtained using an 8 mm biopsy punch. All the PDMS platforms were thoroughly washed with sonication in MilliQ water, detergent and isopropanol. Prior to encapsulation, the PDMS platforms were treated with filtered 5% Plurionic F-127 (Sigma-Aldrich) in PBS for 2 h at 4°C to facilitate hydrogel detachment. The pluronic acid was removed using a filter paper without touching the interior of the platform. The treated PDMS platforms were sterilized in ultraviolet light for 15 min.

### Cell culture

Human immortalized muscle precursor cells were procured from the Institut NeuroMyoGène, Lyon, France (Massenet et al., 2020). Specifically, line control 2-E4 was used with a population-doubling time of 3.55 days and 99.40% of CD56^+^ cells. The cells were cultured in skeletal muscle basal growth medium (SMC-GM, C23060, PromoCell), consisting of supplemental mix (C23060, PromoCell), 1% penicillin-streptomycin (10,000 U ml^−1^, 15140-122, Thermo Fisher Scientific) and 10% fetal bovine serum (10270-106, Thermo Fisher Scientific). For differentiations, the cells were cultured in Dulbecco's modified Eagle medium (DMEM), high glucose, GlutaMAX (Gibco, Thermo Fisher Scientific), 1% v/v penicillin-streptomycin-glutamine (P/S-G, 100×, Gibco, Thermo Fisher Scientific) and 1% v/v insulin-transferrin-selenium-ethanolamine supplement (ITS-X, 100×, Gibco, Thermo Fisher Scientific).

### Tissue fabrication

A previously designed protocol was adopted to fabricate functional skeletal muscle tissues (Tejedera-Villafranca et al., 2023). Briefly, for encapsulation, the human muscle precursor cells were trypsinized and resuspended in skeletal muscle growth medium. The cells were encapsulated at a density of 2.5×10^7^ cells ml^−1^ in 30% v/v Matrigel Growth Factor Reduced (GFR) Basement Membrane Matrix (Corning), 2 U ml^−1^ thrombin from human plasma (Sigma-Aldrich) and 4 mg ml^−1^ fibrinogen from human plasma (Sigma-Aldrich). During hydrogel casting, care was taken to avoid bubbles and cold plasticware was used to prevent polymerization of Matrigel. The mixture was spread as homogenously as possible between the pillars without grazing the surface. After hydrogel introduction, all PDMS platforms were incubated at 37°C for 30 min before adding skeletal muscle growth medium supplemented with 1 mg ml^−1^ of 6-amino-caproic acid (ACA, Sigma-Aldrich). After 48 h, the growth medium was replaced with differentiation medium (DM) supplemented with 1 mg ml^−1^ ACA (DM/ACA). Subsequently, half of the DM/ACA was replenished every 2 days.

### Drug treatments

#### Steroid myopathy induction using DEX

To develop the atrophy model, after 6 days of differentiation, the tissues were treated with 100 µM of DEX (D4902-100MG, Merck Life Science, Germany) in DM/ACA for 24 h as previously reported for C2C12 cells by Shimizu et al., 2017. A stock solution of 10 mM DEX in absolute ethanol was prepared and diluted with DM/ACA to make a final concentration of 100 µM. The same volume of absolute ethanol was used to analyze the effect, if any, of the dissolvent.

#### Evaluation of independent taurine effect

For taurine treatment, 100 mM stock solution of taurine (T0625-25G, Merck Life Science) was prepared in MilliQ water and diluted to 1 mM in DM/ACA. The same volume of MilliQ was used as control. Taurine-supplemented DM/ACA was added on the sixth day of differentiation for 24 h to observe whether the drug had any effect on healthy tissues.

#### Rescue treatment with taurine

To investigate the atrophy amelioration capacity of taurine, two rescue regimes were adopted: (1) entirely replacing the DEX-containing medium with DM/ACA containing 1 mM taurine for another 24 h, or (2) continuing DEX treatment for 48 h and adding 1 mM taurine after 24 h. The final concentrations of DEX and taurine were 50% of their initial values. MilliQ water was used as a control in both cases. Contractile force analysis and immunofluorescence studies were performed on day 8 of differentiation, post treatment.

### Electrical pulse stimulation

Post treatment, the bioengineered skeletal muscle tissues were placed in a 12-well plate with custom-built graphite electrodes connected to a pulse generator (Multifunction Generator WF1974, NF Corporation). An XL S1 cell incubator maintained at 37°C and 5% CO_2_ was used to acquire brightfield images of the plate on top of a Zeiss Axio Observer Z1/7 microscope. The tissues were electrically stimulated to induce both twitch and tetanic contractions. Square pulses of 1 to 50 Hz in an electrical field strength of 1 V mm^−1^ were applied for 10 s with a 10% duty cycle. All experiments were performed with fresh DM/ACA.

#### Video analyses and force measurement

Based on the theory of linear bending, the Euler–Bernoulli's beam-bending equation was used to calculate the force exerted by each tissue. Readings were recorded from both pillars and the average force of contraction was calculated using Fiji/ImageJ.

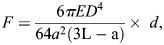


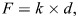


where *F* is the force exerted by the tissue, *E* is the Young's modulus of the PDMS (calculated to be 1.6±0.1 MPa), *D* is the diameter of the post, *a* is the location of the tissue on the pillar, *L* is the height of the post, and *d* is the displacement of the pillar. The spring constant *k* was calculated using the above parameters to be 3.54 N m^−1^ (Fernández-Costa et al., 2023a).

Post stimulation, the tissues were either fixed in 10% formalin solution for 30 min at room temperature (RT) to be cryosectioned for immunofluorescence or frozen in liquid nitrogen for RNA extraction and western blotting.

### Cryosectioning and immunofluorescence

Post fixation in 10% formalin, the tissues were embedded in Optimal Cutting Temperature (OCT) compound (PolyFreeze, Sigma-Aldrich) in plastic Cryomolds^®^ (VWR, PA, USA) using chilled isopentane. Transverse sections (15 µm) were obtained by sectioning with a Leica CM1900 cryostat. The sections were transferred to SuperFrost Plus Adhesion slides (Fisher Scientific) and stored at −20°C.

Prior to staining, the tissue sections were encircled with a PAP pen (ImmEdge, Vector laboratories, CA, USA). PBS was used to hydrate the sections before a 15-min permeabilization step with 0.1% Triton X-100 in PBS (PBS-T). Tissue sections were then blocked for 30-40 min at RT in blocking buffer (0.3% Triton X-100 and 3% donkey serum in PBS) followed by incubation with primary antibodies (see Table S1 for details) in blocking buffer overnight at 4°C. After this incubation, the sections were washed three times with PBS-T (5 min per wash) and incubated with fluorophore-tagged secondary antibodies and phalloidin (Table S1) in blocking buffer for 40-60 min at RT. Sections were then washed three times with PBS-T (5 min per wash) and mounted using VECTASHIELD Plus Mounting Medium with DAPI (Palex). After placing coverslips, the corners of the slides were sealed with a transparent enamel sealant.

#### Imaging and quantitative morphological analyses

Sections were analyzed using brightfield and confocal imaging with ZEISS Axio Observer Z1/7 and ZEISS LSM800 microscopes, respectively. 20× and higher magnifications were used to procure images from the confocal microscope. After acquisition, images were processed and analyzed using Fiji/ImageJ. To calculate myotube diameter, SAA or phalloidin and DAPI channels were used. Images were binarized and the free hand tool was used to mark the boundary for obtaining Ferret's diameter (*n*≥50 per image, at least three images were procured per tissue). The localization of total GCR and FOXO1 content was checked relative to DAPI, phalloidin and SAA signals. Separate channels for the images in Figs 1M, 2M and 3M are shown in Fig. S4. To evaluate colocalization of the GCR with the nucleus, the Fiji/ImageJ plugin JACoP was used (Bolte and Cordelières, 2006; Schindelin et al., 2012). Mander's M1 colocalization coefficients were obtained and compared for different treatment conditions (Manders et al., 1993).

### RNA extraction and qRT-PCR

Sterilized pestles were used to homogenize the tissues in QIAzol reagent (QIAGEN), and the total RNA was extracted using the miRNeasy Micro Kit (QIAGEN) according to the manufacturer's instructions. 1 μg of RNA was digested with DNaseI (Invitrogen) and retrotranscribed with SuperScriptII (Invitrogen) using random hexanucleotides. For each biological replicate, qRT–PCR reactions from 10 ng of cDNA were carried out in triplicate using TaqMan probes (Applied Biosystems; Table S2). *GAPDH* was used as the endogenous control after comparing its stable expression across different treatment regimens (Fig. S6). Thermal cycling was performed using the StepOne Plus RT-PCR System (Applied Biosystems). Relative expression to the endogenous gene and the control group was obtained by the 2^−ΔΔCt^ method. Pairs of samples were compared using a two-tailed unpaired *t*-test (α=0.05), applying Welch's correction when necessary.

### Western blotting

For total protein extraction, samples were homogenized in RIPA buffer (Thermo Fisher Scientific, 10230544) supplemented with protease and phosphatase inhibitor cocktails (Roche Applied Science, 4906837001). Total protein was quantified using a BCA assay kit (Thermo Scientific Pierce, Grand Island, NY, USA, 10741395) following the manufacturer’s instructions. For detection of specific proteins, 25 µg of total protein was denatured for 5 min at 100°C, electrophoresed on 12-15% SDS-PAGE gels and transferred onto 0.45 µm nitrocellulose membranes (Cytiva, Little Chalfont, UK, GE10600002). Membranes were blocked for 1 h at RT with blocking solution containing 5% non-fat dried milk in PBS-T (8 mM Na_2_HPO_4_, 150 mM NaCl, 2 mM KH_2_PO_4_, 3 mM KCl, 0.05% Tween 20, pH 7.4) for non-phosphorylated proteins and 5% bovine serum albumin in PBS-T for phosphorylated proteins. Membranes were incubated with agitation overnight in blocking solution at 4°C with the corresponding primary antibody described in Table S3. Primary antibodies were detected using goat HRP-conjugated anti-rabbit-IgG (1 h, 1:3500, Sigma-Aldrich, A0545) or goat HRP-conjugated anti-mouse-IgG (1:3500, Sigma-Aldrich, B7264) incubated for 1 h at RT. Immunoreactive bands were detected using enhanced chemiluminescence (SuperSignal West Pico PLUS, 34579, Thermo Fisher Scientific) western blotting substrate. Membranes were stripped to remove both the primary and secondary antibodies. For that, membranes were washed with water for 5 min, then incubated with a stripping solution (8 g/l NaOH and 10 g/l SDS in MilliQ water) for 10 min and finally with water for 5 min. The membranes were then blocked again with blocking solution containing 5% non-fat dried milk in PBS-T for 1 h at RT. Finally, the membranes were incubated for 1 h at RT with horseradish peroxidase (HRP)-conjugated anti-GAPDH antibody as a loading control. Images were acquired with an Amersham ImageQuant 800 and were quantified using Fiji/ImageJ software (National Institutes of Health). The corresponding primary antibodies used are described in Table S3. Uncropped blots are shown in Figs S7-S10. In the case of mTOR blots, the upper bands were quantified based on the weight.

### Statistical analysis

Statistical analyses were performed using Prism 9.5 software (GraphPad). All data were tested for normality by using the Shapiro–Wilk test (*P*≥0.05). The comparisons between more than two groups were performed by one-way ANOVA. For two groups, unpaired two-tailed parametric Student's *t*-test with Welch's correction was applied, whereas for non-normal data sets, unpaired two-tailed non-parametric Student's *t*-test with Mann–Whitney U test was performed. In the case of ANOVA, if a significant trend was observed, Tukey's post hoc tests with 95% confidence interval were applied (*P*<0.05).

## Supplementary Material

10.1242/dmm.050540_sup1 Supplementary information

## First Person

This article has an associated First Person interview with the first author of the paper.
