# Supplementary material for: Taurine activates the AKT–mTOR axis to restore muscle mass and contractile strength in human 3D in vitro models of steroid myopathy
Source: Dis Model Mech. 2024 Apr 24;17(4):dmm050540. doi: 10.1242/dmm.050540 (PMC11073513; doi:10.1242/dmm.050540)
Supplement: Supplementary information [file dmm-17-050540-s1.pdf]

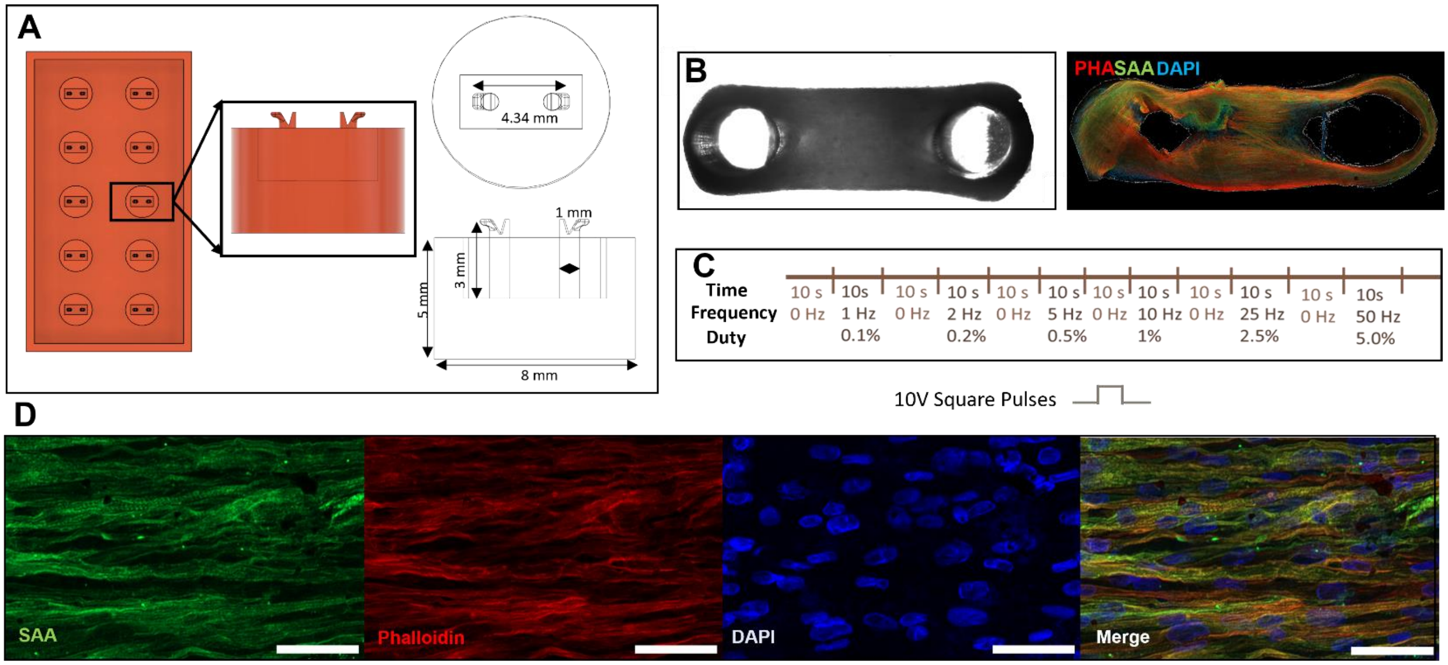

Fig. S1. Fabrication of 3D *in vitro* skeletal muscle tissues. (A) 3D mold design and soft-lithographic process to fabricate PDMS platforms. (B) Representative brightfield and confocal images (stained for α-sarcomeric actinin (SAA), phalloidin (F-actin) and DAPI (nuclei) of a 3D skeletal muscle tissue. (C) Scheme of electric pulse stimulation including frequencies for twitch and tetanic contractions. (D) Representative longitudinal confocal image of a 3D skeletal muscle tissue stained for α-sarcomeric actinin (SAA), phalloidin (F-actin) and DAPI (nuclei). Scale bar = 50 µm.

Disease Models & Mechanisms • Supplementary information

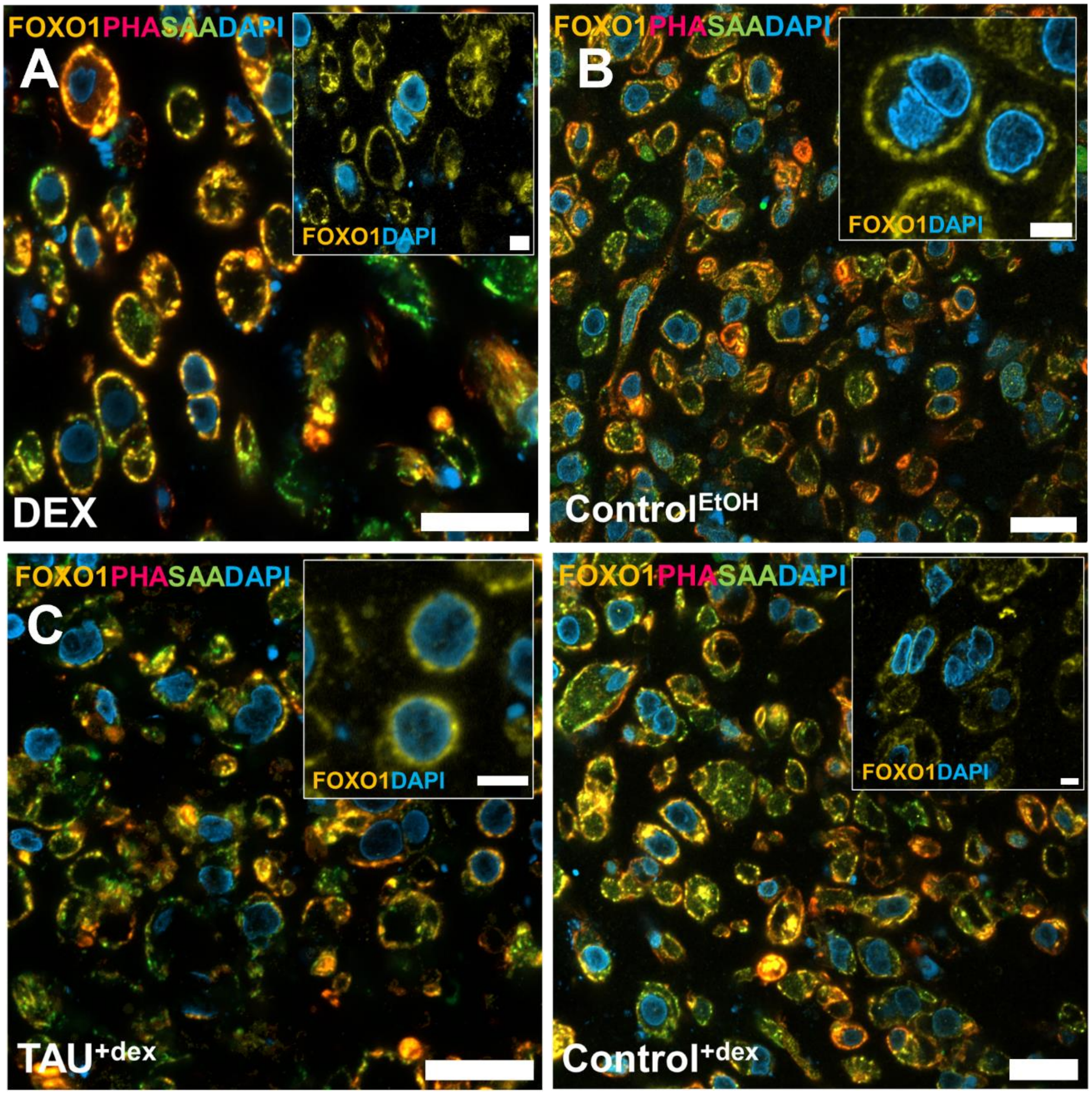

Fig. S2. Immunostaining for FOXO1 in transverse tissue sections stained for FOXO1 (yellow), phalloidin for F-actin (red), α-sarcomeric actinin (green), and DAPI (blue). (A) Tissues treated with 100uM Dexamethasone for 24 hours. (B) Control Tissues for A. (C) Tissues concurrently treated with Dex and Tau (1:1). (D) Control tissues for combinatorial therapy of Dexamethasone with Taurine. Scale bar = 20 µm and 5 µm

Disease Models & Mechanisms • Supplementary information

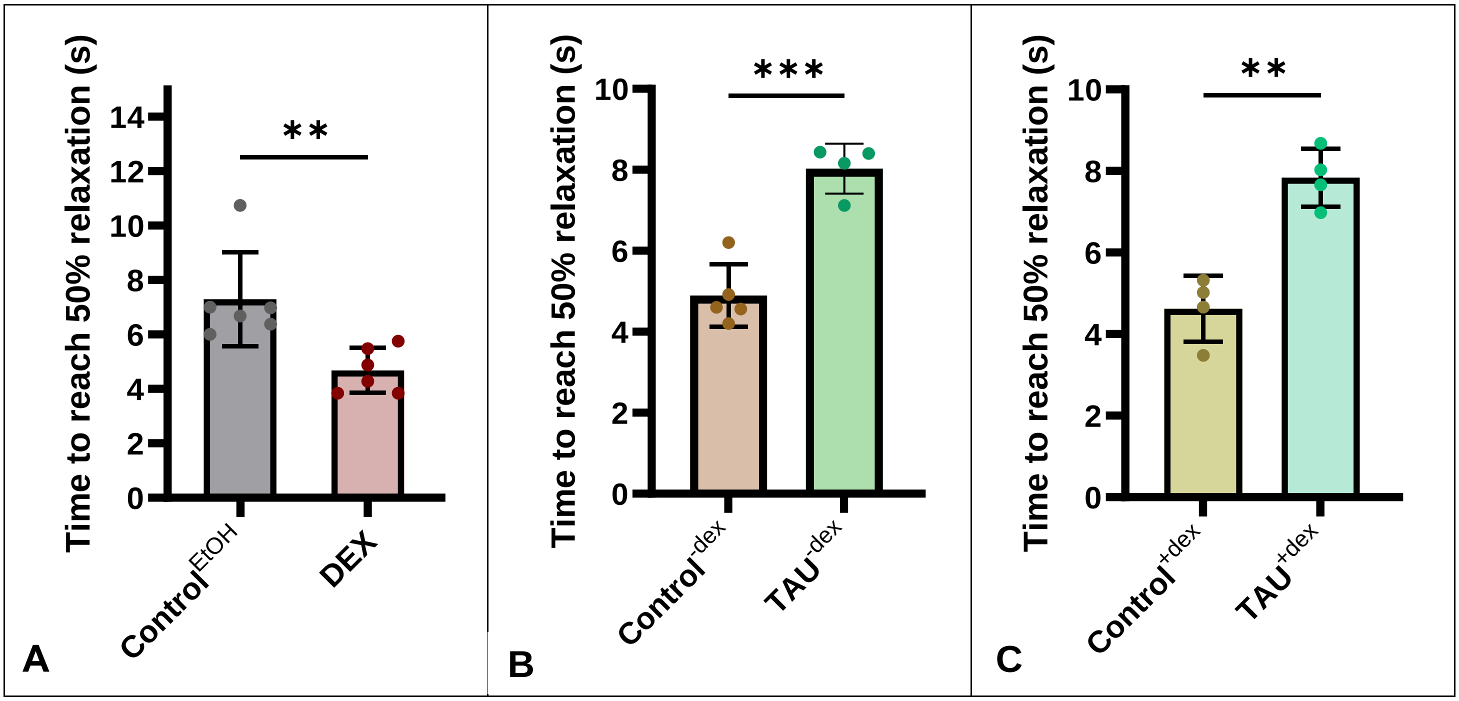

Fig. S3. Quantification of time to reach half relaxation for (A) myopathic tissues and vehicle control, (B) Taurine treated and control myopathic tissues without Dexamethasone, and (C) tissues treated concurrently with Taurine and Dexamethasone (1:1) and control. Statistical analyses: (A) unpaired, non-parametric Student's *t*-test was performed with Mann-Whitney U test (B and C) unpaired, parametric Student's *t*-test with Welch's correction (p **≤ 0.01 ***≤ 0.001).

Disease Models & Mechanisms • Supplementary information

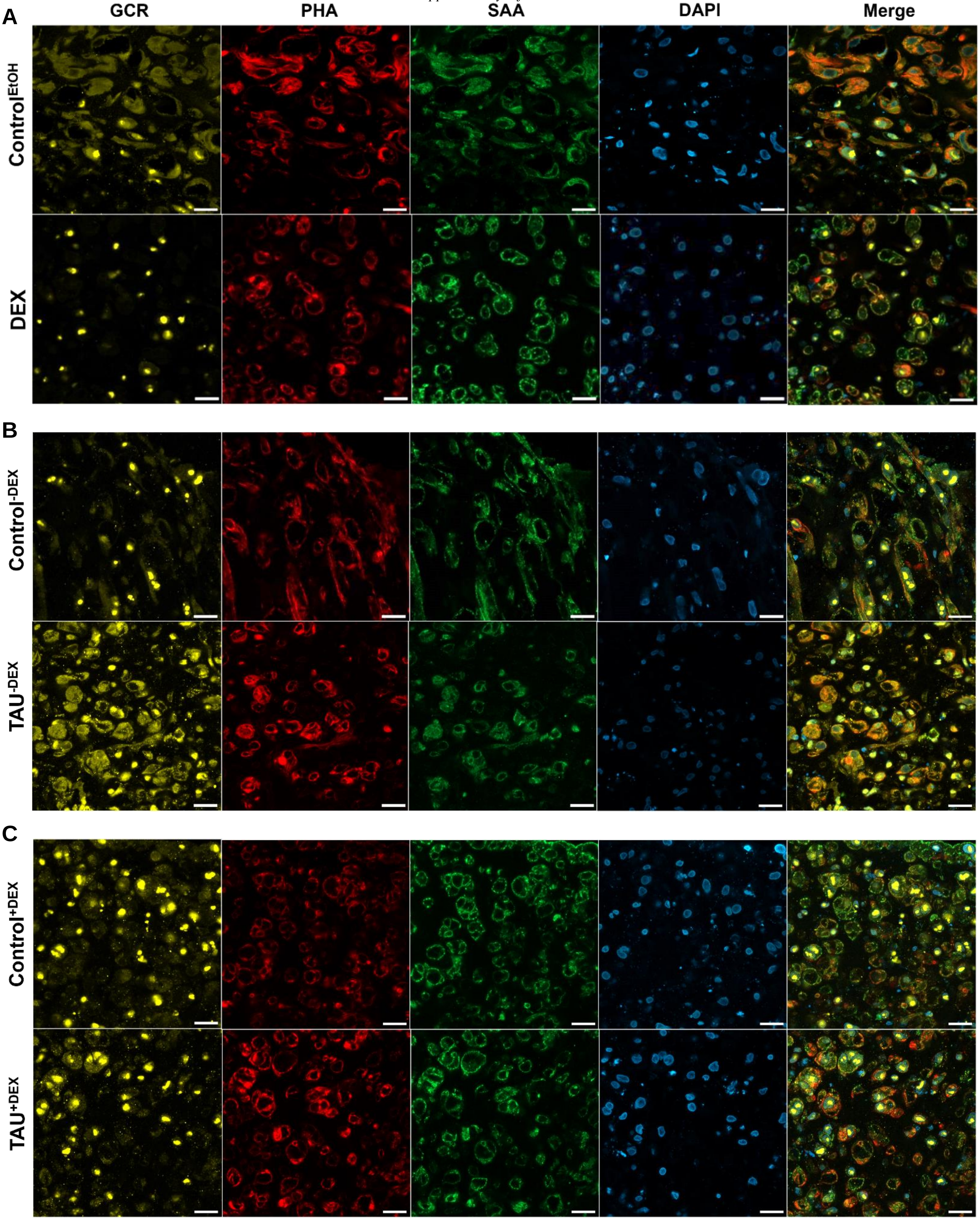

Disease Models & Mechanisms • Supplementary information

Fig. S4. Unmerged immunostaining images for GCR in transverse tissue sections stained for GCR (yellow), phalloidin for F-actin (red), α-sarcomeric actinin (green), and DAPI (blue). (A) Tissues treated with Vehicle (EtOH) and 100uM Dexamethasone for 24 hours. (B) Control and Taurine treated myopathic tissues with Dexamethasone in the system. (C) Tissues concurrently treated with Dex and Tau (1:1). Scale bar = 20 µm. The panels correspond to Figs 1M, 2M and 3M, respectively.

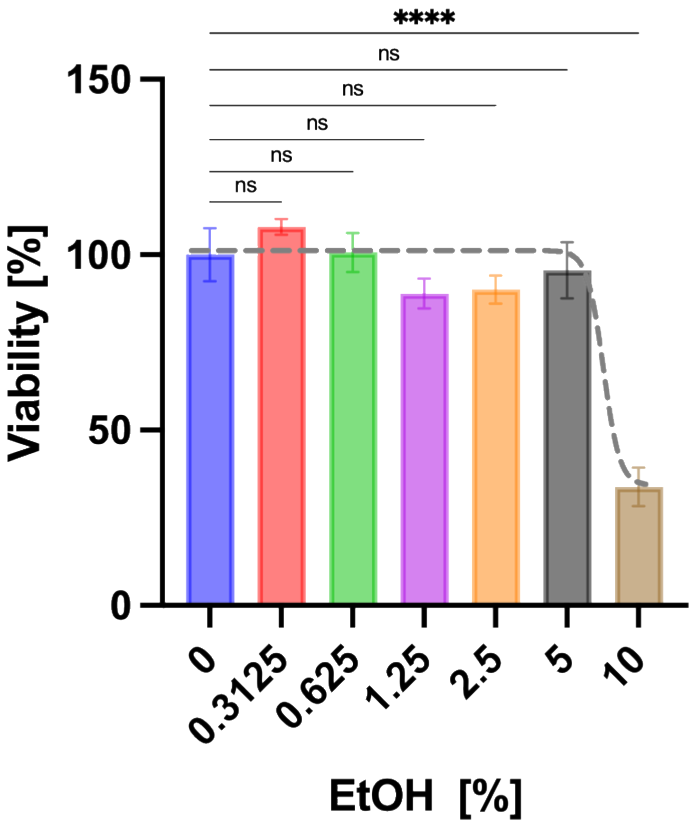

Fig. S5. Dose-response curve of EtOH expressed as percentage viability at percentage (v/v) of EtOH in differentiation medium. Statistical Analysis: one-way ANOVA with Tukey's post-hoc test at 95% confidence interval.

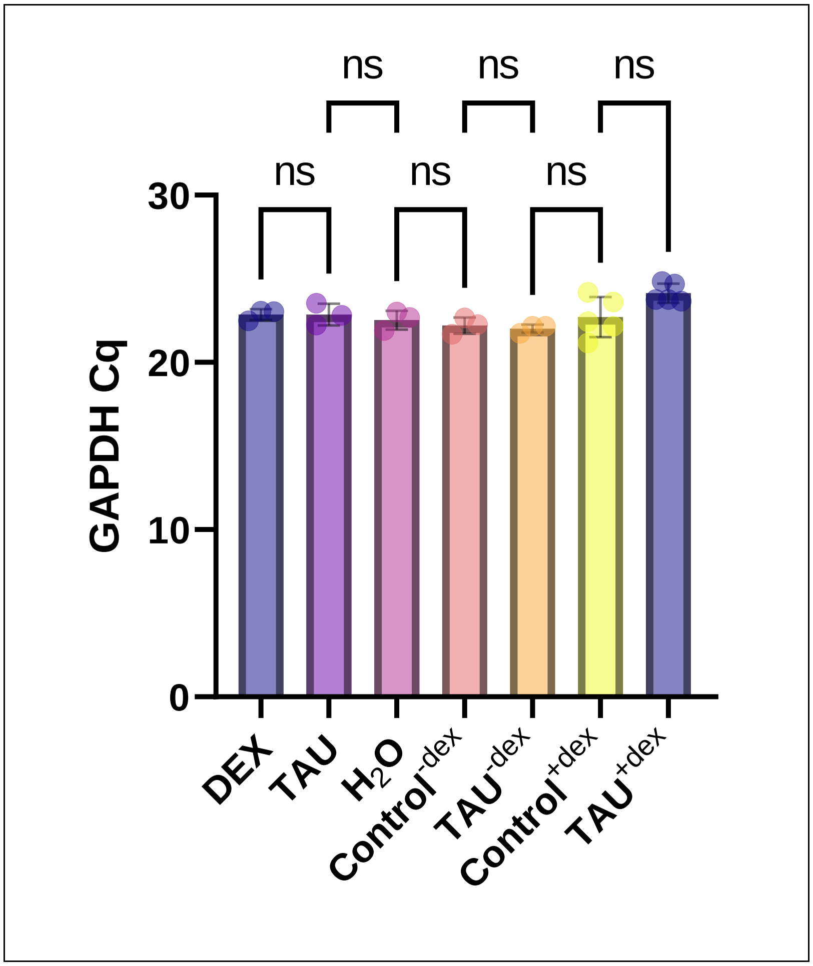

Fig. S6. Stable GAPDH Cq levels across different treatment regimens. Statistical analyses: one-way ANOVA with Tukey's post-hoc test at 95% confidence interval.

Disease Models & Mechanisms • Supplementary information

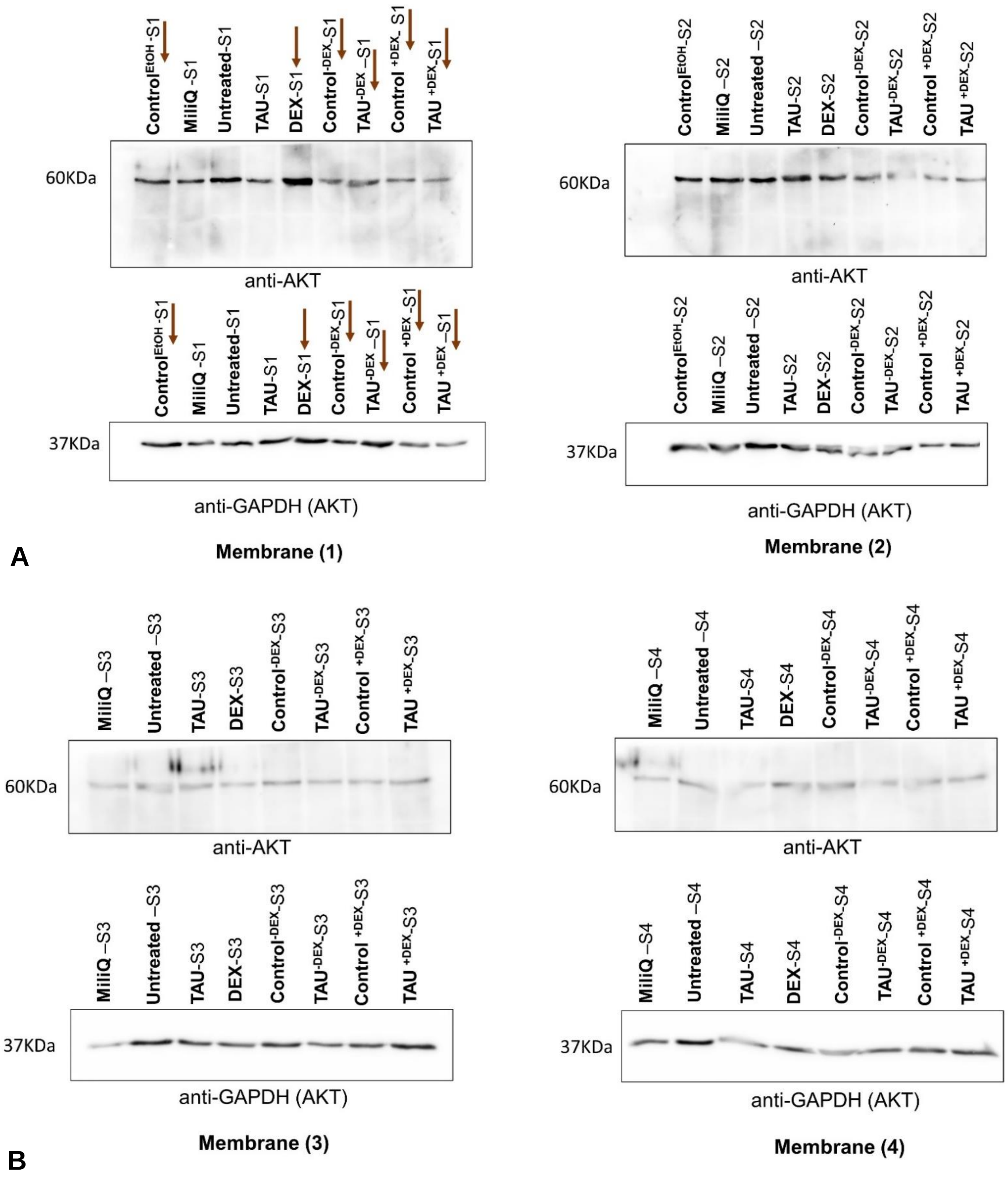

Fig. S7. Uncropped western blots for (A) anti-AKT with corresponding GAPDH bands membranes 1-2, and (B) anti-AKT with corresponding GAPDH bands membranes 3-4, corresponding to Figs 1, 2, 3 and 4. Arrows indicate representative bands shown in the manuscript (Figs 1L, 2L and 3L).

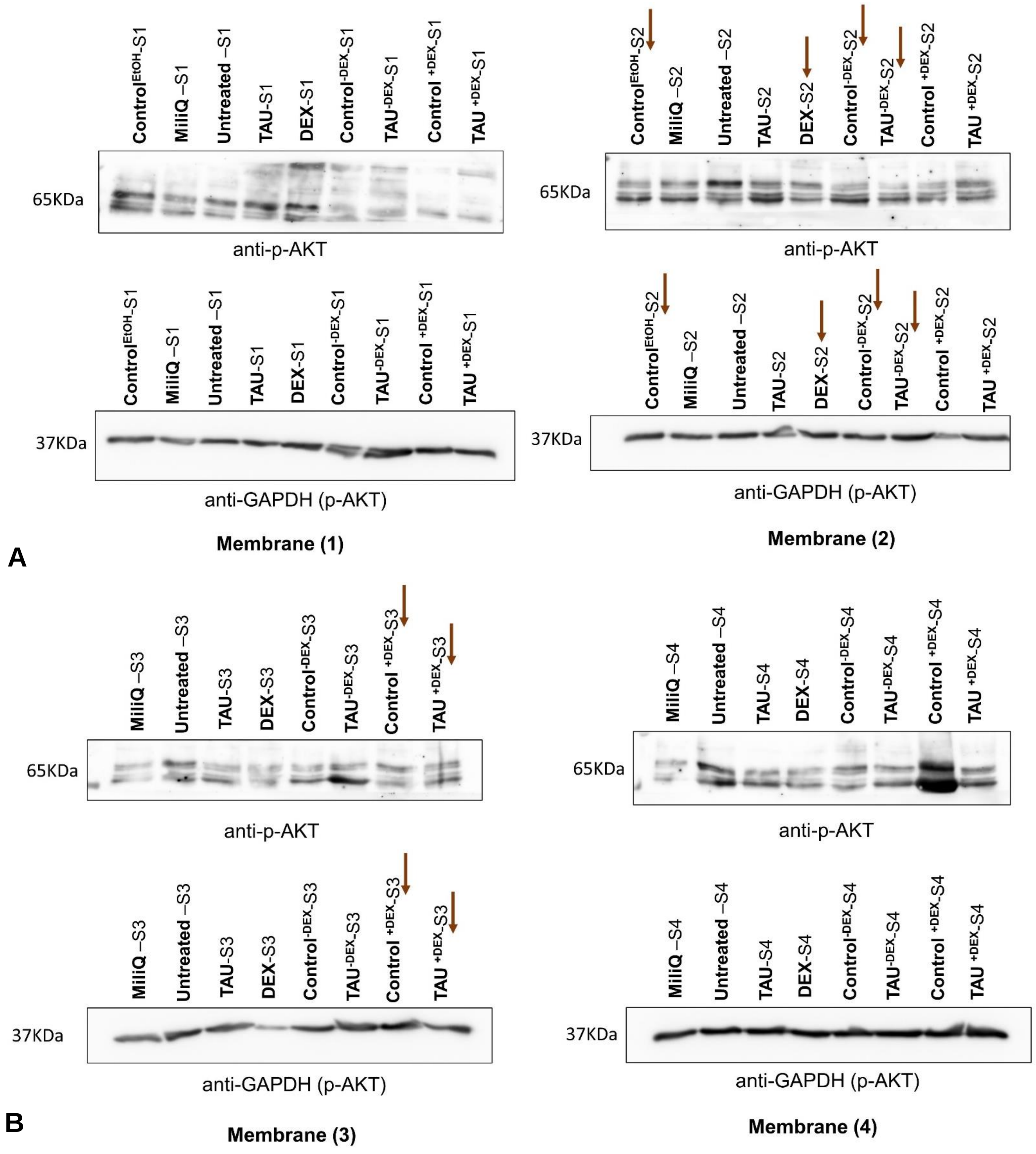

Fig. S8. Uncropped western blots for (A) anti-p-AKT with corresponding GAPDH bands membranes 1-2, and (B) anti-p-AKT with corresponding GAPDH bands membranes 3-4, corresponding to Figs 1, 2, 3 and 4. Arrows indicate representative bands shown in the manuscript (Figs 1L, 2L and 3L).

Disease Models & Mechanisms • Supplementary information

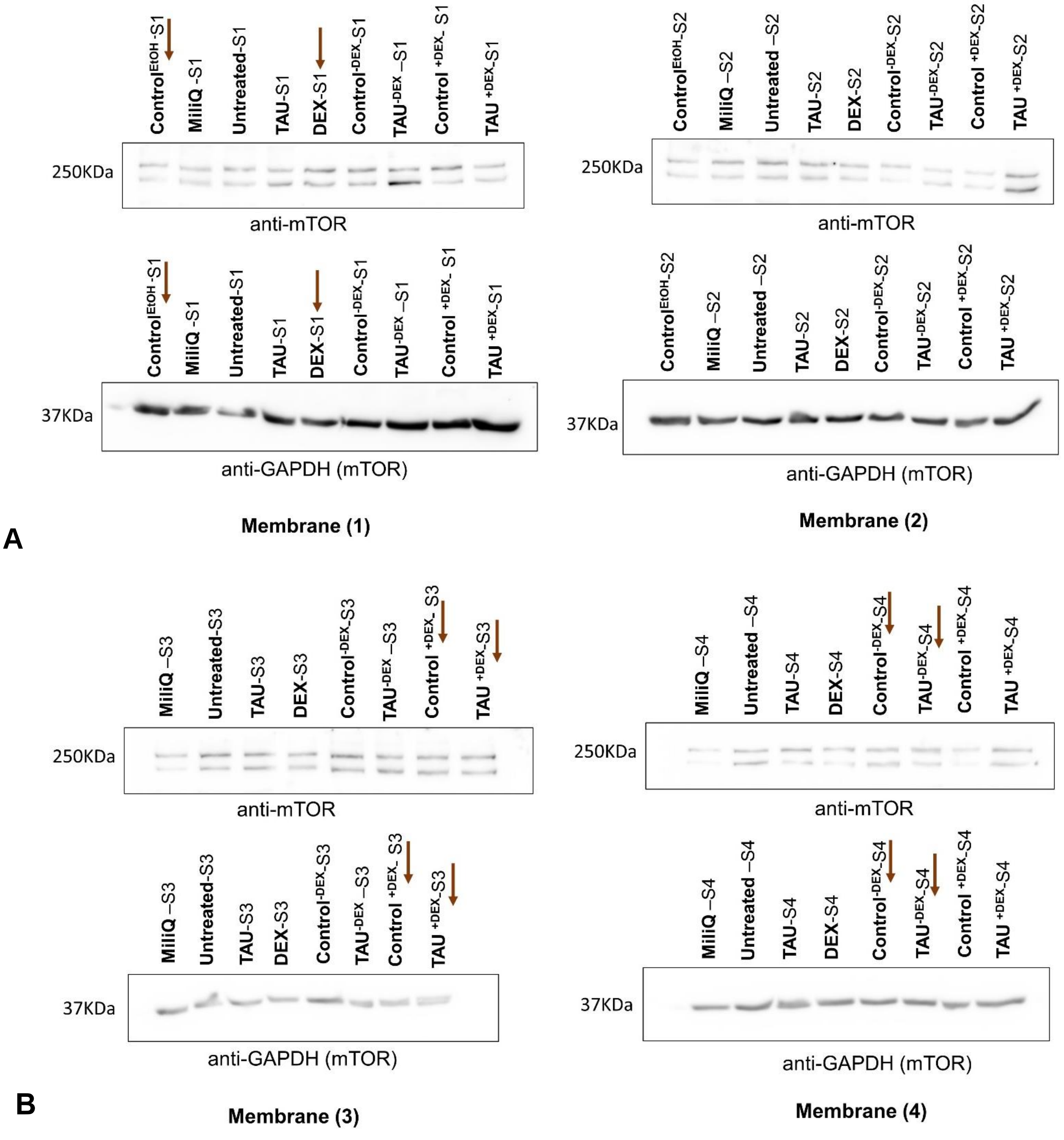

Fig. S9. Uncropped western blots for (A) anti-m-TOR with corresponding GAPDH bands membranes 1-2, and (B) anti-mTOR with corresponding GAPDH bands membranes 3-4, corresponding to Figs 1, 2, 3 and 4. Upper bands were used for mTOR quantification. Arrows indicate representative bands shown in the manuscript (Figs 1L, 2L and 3L).

Disease Models & Mechanisms • Supplementary information

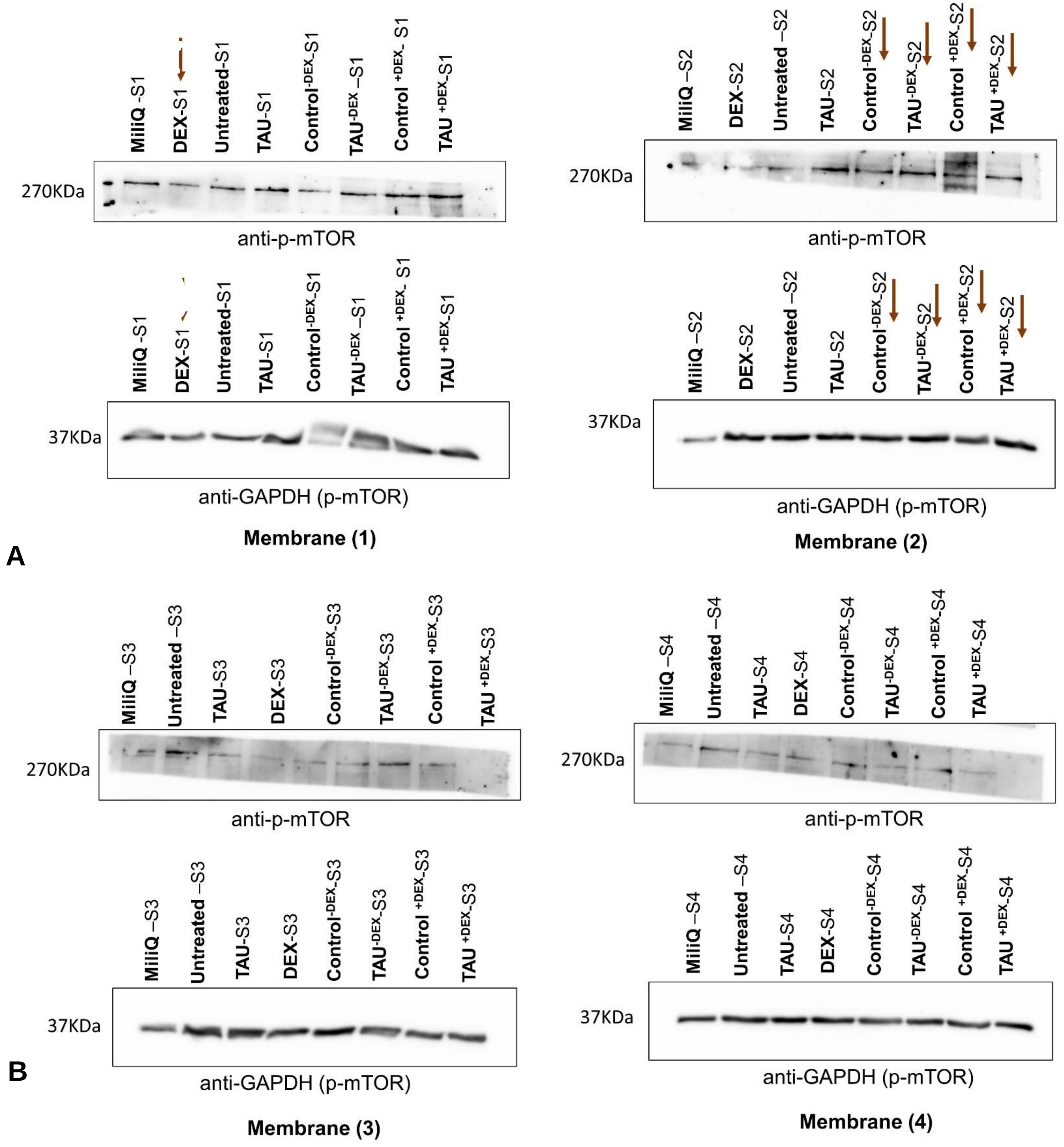

Disease Models & Mechanisms • Supplementary information

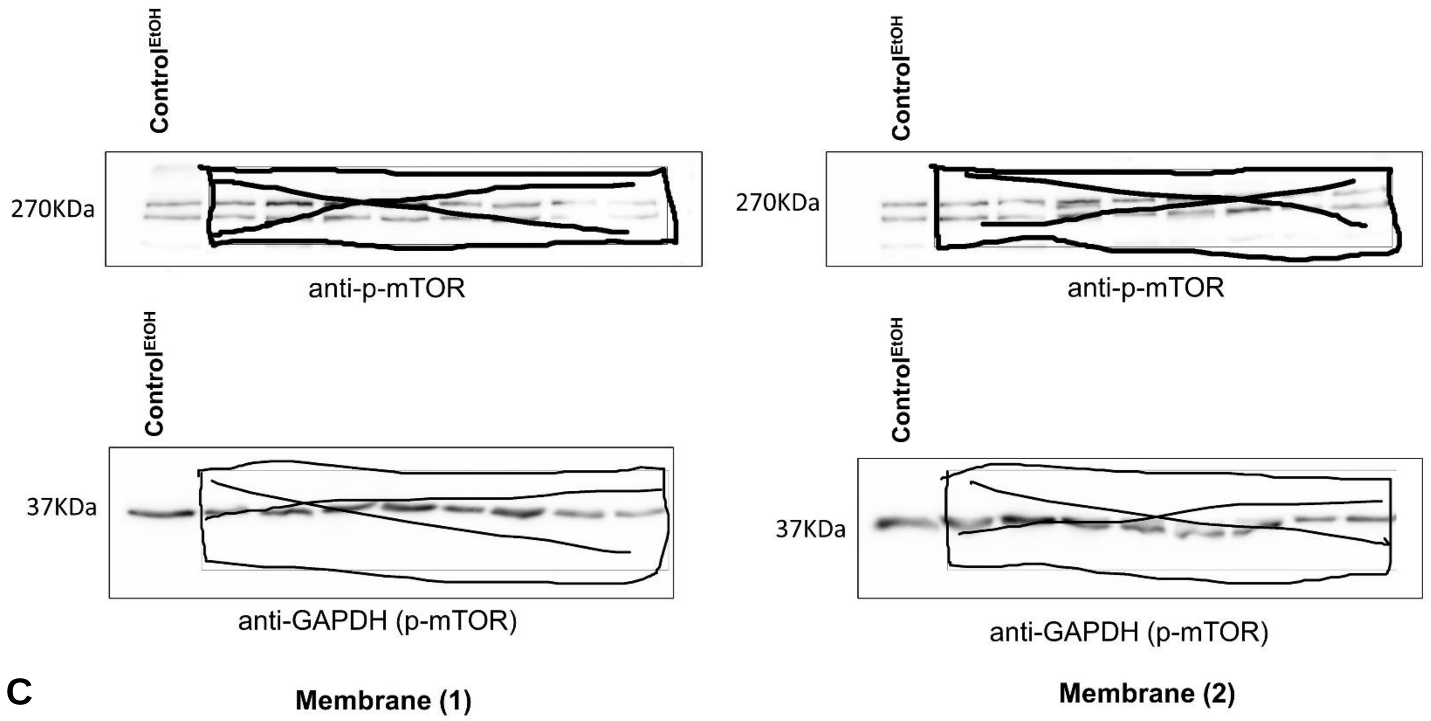

Fig. S10. Uncropped western blots for (A) Anti-p-mTOR with corresponding GAPDH bands membranes 1-2, (B) anti-p-mTOR with corresponding GAPDH bands membranes 3-4, corresponding to Figs 1, 2, 3 and 4, and (C) anti-p-mTOR with corresponding GAPDH bands membranes 1-2 3 for Control^{EtOH}. The GAPDH in these membranes is the same as for AKT. After transfer, the membranes were cut, blocked, and incubated with the corresponding primary antibodies, anti-AKT or anti-p-mTOR, given their markedly different sizes. Arrows indicate representative bands shown in the manuscript (Figs 1L, 2L and 3L). p-mTOR blots for Dex and Control^{EtOH} and their quantification was not shown in the manuscript (Fig 1K-L) because the blots were on different membranes. Regardless, the quantification indicated significant difference as well.

Table S1. Antibodies and probes

Name	Target	Working Dilution	Company
Monoclonal mouse Anti-α-Actinin (Sarcomeric)	Sarcomeric α-Actinin	1:200	Merck Life Science #A7811
Alexa Fluor™ 594 Phalloidin	F-actin	1:400	Invitrogen #A12381
Polyclonal Rabbit Glucocorticoid Receptor Primary Antibody	Glucocorticoid receptor	1:100	Invitrogen #PA1-512
Polyclonal donkey anti-mouse IgG, Alexa Fluor™ 488- conjugated secondary antibody.	Mouse IgG	1:200	Invitrogen #A21202
Polyclonal donkey anti-rabbit IgG, Alexa Fluor™ 647- conjugated secondary antibody.	Rabbit IgG	1:200	Invitrogen #A32795

Table S2. Primer sequences for gene expression analysis

Gene Name	TaqManTM Gene Expression Assay (FAM) ID	Company
FOXO1	Hs00231106_m1	Thermofisher Scientific
FOXO3, FOXO3B	Hs00921424_m1	Thermofisher Scientific
GAPDH	Hs99999905_m1	Thermofisher Scientific
AKT1	Hs00178289_m1	Thermofisher Scientific
TRIM63	Hs00822397_m1	Thermofisher Scientific
FBXO32	Hs01041408_m1	Thermofisher Scientific

Table S3. Antibodies for western blot

Protein name	Host species	Antibody dilution	Size	Reference ID	Company
Phospho-mTOR (Ser2448)	Mouse	1:3500	250-279 kDa	67778-1-IG	Proteintech
mTOR	Mouse	1:2000	250 kDa	66888-1-IG	Proteintech
Phospho-Akt (Ser473)	Rabbit	1:1000	60-65 kDa	#9271	Cell Signaling Technology (Danvers, MA, USA)
AKT	Rabbit	1:1000	60 kDa	#9272	Cell Signaling Technology (Danvers, MA, USA)
GAPDH-HRP	Mouse	1:3500	37kDa	Sc-365062	Santa Cruz, Dallas (Texas, USA)

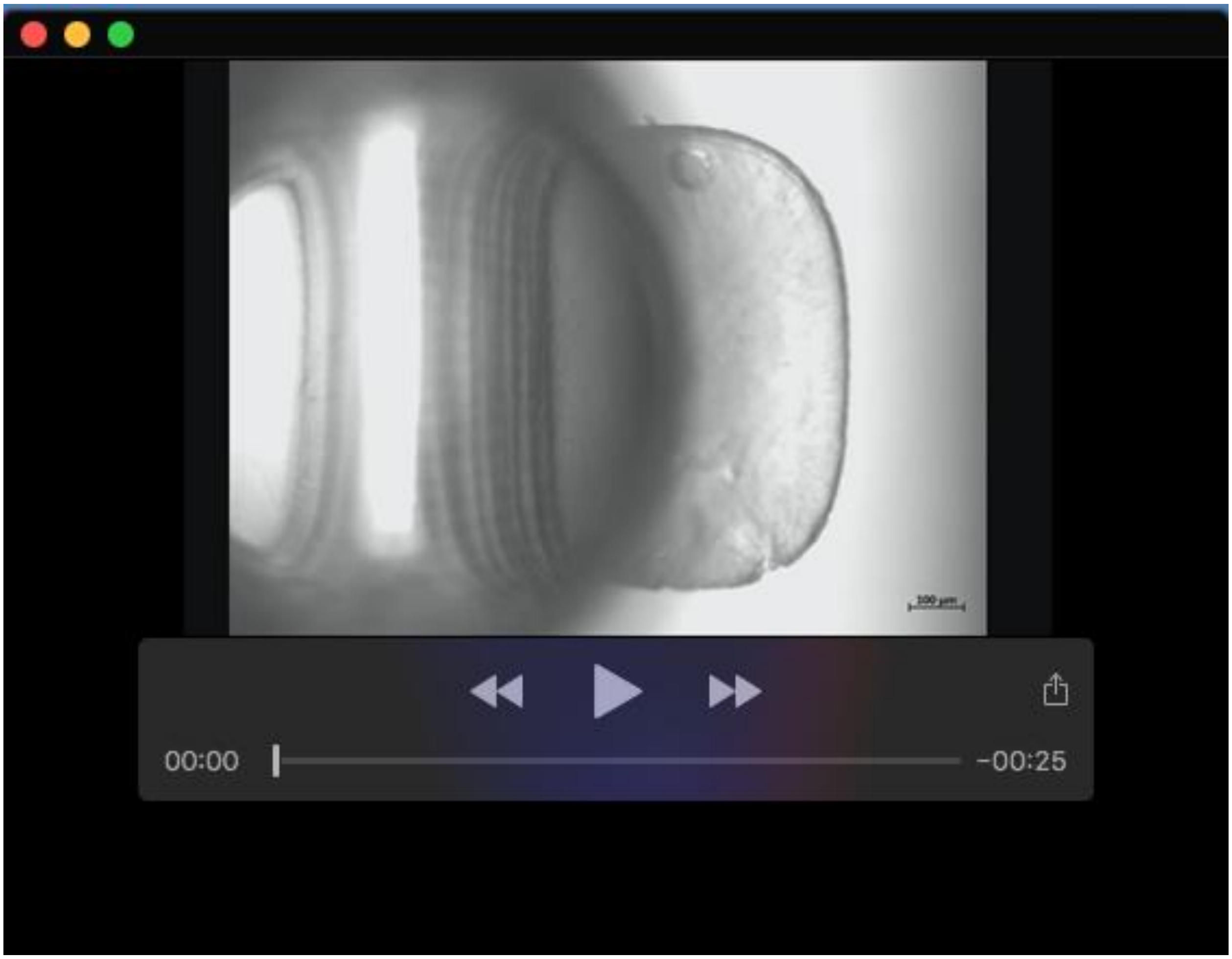

Movie 1. Representative brightfield video of 3D skeletal muscle tissue responding to the electric pulse stimulation scheme as shown in Fig. S1C.
